# Shannon Entropy Estimation in ∞-Alphabets from Convergence Results: Studying Plug-In Estimators

**DOI:** 10.3390/e20060397

**Published:** 2018-05-23

**Authors:** Jorge F. Silva

**Affiliations:** Information and Decision System Group, Department of Electrical Engineering, Universidad de Chile, Av. Tupper 2007, Santiago 7591538, Chile; josilva@ing.uchile.cl

**Keywords:** Shannon entropy estimation, countably infinite alphabets, entropy convergence results, statistical learning, histogram-based estimators, data-driven partitions, strong consistency, rates of convergence

## Abstract

This work addresses the problem of Shannon entropy estimation in countably infinite alphabets studying and adopting some recent convergence results of the entropy functional, which is known to be a discontinuous function in the space of probabilities in ∞-alphabets. Sufficient conditions for the convergence of the entropy are used in conjunction with some deviation inequalities (including scenarios with both finitely and infinitely supported assumptions on the target distribution). From this perspective, four plug-in histogram-based estimators are studied showing that convergence results are instrumental to derive new strong consistent estimators for the entropy. The main application of this methodology is a new data-driven partition (plug-in) estimator. This scheme uses the data to restrict the support where the distribution is estimated by finding an optimal balance between estimation and approximation errors. The proposed scheme offers a consistent (distribution-free) estimator of the entropy in ∞-alphabets and optimal rates of convergence under certain regularity conditions on the problem (finite and unknown supported assumptions and tail bounded conditions on the target distribution).

## 1. Introduction

Shannon entropy estimation has a long history in information theory, statistics, and computer science [1]. Entropy and related information measures (conditional entropy and mutual information) have a fundamental role in information theory and statistics [2,3] and, as a consequence, it has found numerous applications in learning and decision making tasks [4,5,6,7,8,9,10,11,12,13,14,15]. In many of these contexts, distributions are not available and the entropy needs to be estimated from empirical data. This problem belongs to the category of scalar functional estimation that has been thoroughly studied in non-parametric statistics.

Starting with the finite alphabet scenario, the classical plug-in estimator (i.e., the empirical distribution evaluated on the functional) is well known to be consistent, minimax optimal, and asymptotically efficient [16] (Section 8.7–8.9). More recent research has focused on looking at the so-called large alphabet (or large dimensional) regime, meaning a non-asymptotic under-sampling regime where the number of samples *n* is on the order of, or even smaller than, the size of the alphabet denoted by *k*. In this context, it has been shown that the classical plug-in estimator is sub-optimal as it suffers from severe bias [17,18]. For characterizing optimality in this high dimensional context, a non-asymptotic minimax mean square error analysis (under a finite *n* and *k*) has been explored by several authors [17,18,19,20,21] considering the minimax risk

$$R^{*}\left(k,n\right)=\underset{\hat{H}\left(\cdot \right)}{\inf}\underset{\mu \in P(k)}{\sup}E_{X_{1},\ldots X_{n}∼\mu^{n}}\left\{{\left(\hat{H}\left(X_{1},\ldots ,X_{n}\right)-H\left(\mu \right)\right)}^{2}\right\}$$

where P(k) denotes the collection of probabilities on $\left[k\right]\equiv \left\{1,\ldots ,k\right\}$ and H(μ) is the entropy of μ (details in Section 2). Paninski [19] first showed that it was possible to construct an entropy estimator that uses a sub-linear sampling size to achieve minimax consistency when *k* goes to infinity, in the sense that there is a sequence $\left(n_{k}\right)=o\left(k\right)$ where $R^{*}\left(k,n_{k}\right)⟶0$ as *k* goes to infinity. A set of results by Valiant et al. [20,21] shows that the optimal scaling of the sampling size with respect to *k* is *O*(*k*/log(k)), to achieve the aforementioned asymptotic consistency for entropy estimation. A refined set of results for the complete characterization of $R^{*}\left(k,n\right)$, the specific scaling of the sampling complexity, and the achievability of the obtained minimax $L_{2}$ risk for the family $\left\{P(k):k\geq 1\right\}$ with practical estimators have been presented in [17,18]. On the other hand, it is well-known that the problem of estimating the distribution (consistently in total variation) in finite alphabets requires a sampling complexity that scales as O(k) [22]. Consequently, in finite alphabets the task of entropy estimation is simpler than estimating the distribution in terms of sampling complexity. These findings are consistent with the observation that the entropy is a continuous functional of the space of distributions (in the total variational distance sense) for the finite alphabet case [2,23,24,25].

### 1.1. The Challenging Infinite Alphabet Learning Scenario

In this work, we are interested in the countably infinite alphabet scenario, i.e., on the estimation of the entropy when the alphabet is countably infinite and we have a finite number of samples. This problem can be seen as an infinite dimensional regime as the size of the alphabet goes unbounded and *n* is kept finite for the analysis, which differs from the large dimensional regime mentioned above. As argued in [26] (Section IV), this is a challenging non-parametric learning problem because some of the finite alphabet properties of the entropy do not extend to this infinite dimensional context. Notably, it has been shown that the Shannon entropy is not a continuous functional with respect to the total variational distance in infinite alphabets [24,26,27]. In particular, Ho et al. [24] (Theorem 2) showed concrete examples where convergence in $\chi^{2}$-divergence and in direct information divergence (I-divergence) of a set of distributions to a limit, both stronger than total variational convergence [23,28], do not imply the convergence of the entropy. In addition, Harremoës [27] showed the discontinuity of the entropy with respect to the reverse I-divergence [29], and consequently, with respect to the total variational distance (the distinction between reverse and direct I-divergence was pointed out in the work of Barron et al. [29]). In entropy estimation, the discontinuity of the entropy implies that the minimax mean square error goes unbounded, i.e.,

$$R_{n}^{*}=\underset{\hat{H}\left(\cdot \right)}{\inf}\underset{\mu \in H(X)}{\sup}E_{X_{1},\ldots X_{n}∼\mu^{n}}\left\{{\left(\hat{H}\left(X_{1},\ldots ,X_{n}\right)-H\left(\mu \right)\right)}^{2}\right\}=\infty ,$$

where H(X) denotes the family of finite entropy distribution over the countable alphabet set X (the proof of this result follows from [26] (Theorem 1) and the argument is presented in Appendix A). Consequently, there is no universal minimax consistent estimator (in the mean square error sense) of the entropy over the family of finite entropy distributions.

Considering a sample-wise (or point-wise) convergence to zero of the estimation error (instead of the minimax expected error analysis mentioned above), Antos et al. [30] (Theorem 2 and Corollary 1) show the remarkable result that the classical plug-in estimate is strongly consistent and consistent in the mean square error sense for any finite entropy distribution (point-wise). Then, the classical plug-in entropy estimator is universal, meaning that the convergence to the right limiting value H(μ) is achieved almost surely despite the discontinuity of the entropy. Moving on the analysis of the (point-wise) rate of convergence of the estimation error, Antos et al. [30] (Theorem 3) present a finite length lower bound for the error of any arbitrary estimation scheme, showing as a corollary that no universal rate of convergence (to zero) can be achieved for entropy estimation in infinite alphabets [30] (Theorem 4). Finally, constraining the problem to a family of distributions with specific power tail bounded conditions, Antos et al. [30] (Theorem 7) present a finite length expression for the rate of convergence of the estimation error of the classical plug-in estimate.

### 1.2. From Convergence Results to Entropy Estimation

In view of the discontinuity of the entropy in ∞-alphabets [24] and the results that guarantee entropy convergence [25,26,27,31], this work revisits the problem of point-wise almost-sure entropy estimation in ∞-alphabets from the perspective of studying and applying entropy convergence results and their derived bounds [25,26,31]. Importantly, entropy convergence results have established concrete conditions on both the limiting distribution μ and the way a sequence of distributions $\left\{\mu_{n}:n\geq 0\right\}$ converges to μ such that $\lim_{n\to \infty }H\left(\mu_{n}\right)=H\left(\mu \right)$ is satisfied. The natural observation that motivates this work is that consistency is basically a convergence to the true entropy value that happens with probability one. Then our main conjecture is that putting these conditions in the context of a learning task, i.e., where $\left\{\mu_{n}:n\geq 0\right\}$ is a random sequence of distributions driven by the classical empirical process, will offer the possibility to study a broad family of plug-in estimators with the objective to derive new strong consistency and rates of convergence results. On the practical side, this work proposes and analizes a data-driven histogram-based estimator as a key learning scheme, since this approach offers the flexibility to adapt to learning task when appropriate bounds for the estimation and approximation errors are derived.

### 1.3. Contributions

We begin revisiting the classical plug-in entropy estimator considering the relevant scenario where μ (the unknown distribution that produces the i.i.d. samples) has a finite but arbitrary large and unknown support. This is declared to be a challenging problem by Ho and Yeung [26] (Theorem 13) because of the discontinuity of the entropy. Finite-length (non-asymptotic) deviation inequalities and intervals of confidence are derived extending the results presented in [26] (Section IV). From this, it is shown that the classical plug-in estimate achieves optimal rates of convergence. Relaxing the finite support restriction on μ, two concrete histogram-based plug-in estimators are presented, one built upon the celebrated Barron-Györfi-van der Meulen histogram-based approach [29,32,33]; and the other on a data-driven partition of the space [34,35,36]. For the Barron plug-in scheme, almost-sure consistency is shown for entropy estimation and distribution estimation in direct I-divergence under some mild support conditions on μ. For the data-driven partition scheme, the main context of application of this work, it is shown that this estimator is strongly consistent distribution-free, matching the universal result obtained for the classical plug-in approach in [30]. Furthermore, new almost-sure rates of convergence results (in the estimation error) are obtained for distributions with finite but unknown support and for families of distributions with power and exponential tail dominating conditions. In this context, our results show that this adaptive scheme has a concrete design solution that offers very good convergence rate of the overall estimation error, as it approaches the rate $O(1/\sqrt{n})$ that is considered optimal for the finite alphabet case [16]. Importantly, the parameter selection of this scheme relies on, first, obtaining expressions to bound the estimation and approximation errors and, second, finding the optimal balance between these two learning errors.

### 1.4. Organization

The rest of the paper is organized as follows. Section 2 introduces some basic concepts, notation, and summarizes the main entropy convergence results used in this work. Section 3, Section 4 and Section 5 state and elaborate the main results of this work. Discussion of the results and final remarks are given in Section 6. The technical derivation of the main results are presented in Section 7. Finally, proofs of auxiliary results are relegated to the Appendix Section.

## 2. Preliminaries

Let X be a countably infinite set and let P(X) denote the collection of probability measures in X. For μ and *v* in P(X), and μ absolutely continuous with respect to *v* (i.e., μ≪v), $\frac{d\mu }{dv}\left(x\right)$ denotes the *Radon-Nikodym* (RN) derivative of μ with respect to *v*. Every μ∈P(X) is equipped with its probability mass function (pmf) that we denote by $f_{\mu }\left(x\right)\equiv \mu \left(\left\{x\right\}\right)$, ∀x∈X. Finally, for any μ∈P(X), $A_{\mu }\equiv \left\{x\in X:f_{\mu }\left(x\right)>0\right\}$ denotes its support and

(1) $$F\left(X\right)\equiv \left\{\mu \in P\left(X\right):\left|A_{\mu }\right|<\infty \right\}$$

denotes the collection of probabilities with finite support.

Let μ and *v* be in P(X), then the total variation distance of μ and *v* is given by [28]

(2) $$V\left(\mu ,v\right)\equiv \underset{A\in 2^{X}}{\sup}\left|v(A)-\mu (A)\right|,$$

where $2^{X}$ denotes the subsets of X. The Kullback–Leibler divergence or I-divergence of μ with respect to *v* is given by

(3) $$D\left(\mu ||v\right)\equiv \sum_{x\in A_{\mu }}f_{\mu }\left(x\right)\log\frac{f_{\mu }\left(x\right)}{f_{v}\left(x\right)}\geq 0,$$

when μ≪v, while D(μ||v) is set to infinite, otherwise [37].

The Shannon entropy of μ∈P(X) is given by [1,2,38]:

(4) $$H\left(\mu \right)\equiv -\sum_{x\in A_{\mu }}f_{\mu }\left(x\right)\log f_{\mu }\left(x\right)\geq 0.$$

In this context, let H(X)⊂P(X) be the collection of probabilities where (4) is finite, let AC(X|v)⊂P(X) denote the collection of probabilities absolutely continuous with respect to v∈P(X), and let H(X|v)⊂AC(X|v) denote the collection of probabilities where (3) is finite for v∈P(X).

Concerning convergence, a sequence $\left\{\mu_{n}:n\in N\right\}\subset P\left(X\right)$ is said to converge in total variation to μ∈P(X) if

(5) $$\lim_{n\to \infty }V\left(\mu_{n},\mu \right)=0.$$

For countable alphabets, ref. [31] (Lemma 3) shows that the convergence in total variation is equivalent to the weak convergence, which is denoted here by $\mu_{n}\Rightarrow \mu$, and the point-wise convergence of the pmf’s. Furthermore, from (2), the convergence in total variation implies the uniform convergence of the pmf’s, i.e, $\lim_{n\to \infty }\sup_{x\in X}\left|\mu_{n}\left(\left\{x\right\}\right)-\mu \left(\left\{x\right\}\right)\right|=0$. Therefore, in this countable case, all the four previously mentioned notions of convergence are equivalent: total variation, weak convergence, point-wise convergence of the pmf’s, and uniform convergence of the pmf’s.

We conclude with the convergence in I-divergence introduced by Barron et al. [29]. It is said that $\left\{\mu_{n}:n\in N\right\}$ converges to μ in direct and in reverse I-divergence if $\lim_{n\to \infty }D(\mu ||\mu_{n})=0$ and $\lim_{n\to \infty }D(\mu_{n}\left||\mu \right)=0$, respectively. From Pinsker’s inequality [39,40,41], the convergence in I-divergence implies the weak convergence in (5), where it is known that the converse result is not true [27].

### 2.1. Convergence Results for the Shannon Entropy

The discontinuity of the entropy in ∞-alphabets raises the problem of finding conditions under which convergence of the entropy can be obtained. On this topic, Ho et al. [26] have studied the interplay between entropy and the total variation distance, specifying conditions for convergence by assuming a finite support on the involved distributions. On the other hand, Harremoës [27] (Theorem 21) obtained convergence of the entropy by imposing a power dominating condition [27] (Definition 17) on the limiting probability measure μ for all the sequences $\left\{\mu_{n}:n\geq 0\right\}$ converging in reverse I-divergence to μ [29]. More recently, Silva et al. [25] have addressed the entropy convergence studying a number of new settings that involve conditions on the limiting measure μ, as well as the way the sequence $\left\{\mu_{n}:n\geq 0\right\}$ converges to μ in the space of distributions. These results offer sufficient conditions where the entropy evaluated in a sequence of distributions converges to the entropy of its limiting distribution and, consequently, the possibility of applying these when analyzing plug-in entropy estimators. The results used in this work are summarized in the rest of this section.

Let us begin with the case when μ∈F(X), i.e., when the support of the limiting measure is finite and unknown.

**Proposition** **1.** Let us assume that μ∈F(X) and $\left\{\mu_{n}:n\in N\right\}\subset \mathrm{AC}\left(X|\mu \right)$. If $\mu_{n}\Rightarrow \mu$, then $\lim_{n\to \infty }D(\mu_{n}\left||\mu \right)=0$ and $\lim_{n\to \infty }H\left(\mu_{n}\right)=H\left(\mu \right)$.

This result is well-known because when $A_{\mu_{n}}\subset A_{\mu }$ for all *n*, the scenario reduces to the finite alphabet case, where the entropy is known to be continuous [2,23]. Since we obtain two inequalities that are used in the following sections, a simple proof is provided here.

**Proof.**  μ and $\mu_{n}$ belong to H(X) from the finite-supported assumption. The same argument can be used to show that $D(\mu_{n}||\mu )<\infty$, since $\mu_{n}\ll \mu$ for all *n*. Let us consider the following identity:

(6) $$H\left(\mu \right)-H\left(\mu_{n}\right)=\sum_{x\in A_{\mu }}\left(f_{\mu_{n}}\left(x\right)-f_{\mu }\left(x\right)\right)\log f_{\mu }\left(x\right)+D(\mu_{n}||\mu ).$$

The first term on the right hand side (RHS) of (6) is upper bounded by $M_{\mu }\cdot V\left(\mu_{n},\mu \right)$ where

(7) $$M_{\mu }=\log\frac{1}{m_{\mu }}\equiv \underset{x\in A_{\mu }}{\sup}\left|\log\mu (\left\{x\right\})\right|<\infty .$$

For the second term, we have that

(8) $$\begin{array}{cc} D(\mu_{n}||\mu ) & \leq \log e\cdot \sum_{x\in A_{\mu_{n}}}f_{\mu_{n}}\left(x\right)\left|\frac{f_{\mu_{n}}\left(x\right)}{f_{\mu }\left(x\right)}-1\right|\\ & \leq \frac{\log e}{m_{\mu }}\cdot \underset{x\in A_{\mu }}{\sup}\left|f_{\mu_{n}}\left(x\right)-f_{\mu }\left(x\right)\right|\leq \frac{\log e}{m_{\mu }}\cdot V\left(\mu_{n},\mu \right). \end{array}$$

and, consequently,

(9) $$\begin{array}{c} \left|H\left(\mu \right)-H\left(\mu_{n}\right)\right|\leq \left[M_{\mu }+\frac{\log e}{m_{\mu }}\right]\cdot V\left(\mu_{n},\mu \right). \end{array}$$

☐

Under the assumptions of Proposition 1, we note that the reverse I-divergence and the entropy difference are bounded by the total variation by (8) and (9), respectively. Note, however, that these bounds are a distribution-dependent function of $m_{\mu }$($M_{\mu }$) in (7) (it is direct to show that $m_{\mu }\left(M_{\mu }\right)<\infty$ if, and only if, μ∈F(X)). The next result relaxes the assumption that $\mu_{n}\ll \mu$ and offers a necessary and sufficient condition for the convergence of the entropy.

**Lemma** **1.** *Ref. [25] (Theorem 1) Let μ∈F(X) and $\left\{\mu_{n}:n\in N\right\}\subset F\left(X\right)$. If $\mu_{n}\Rightarrow \mu$, then there exists N>0 such that $\mu \ll \mu_{n}$∀n≥N, and*

$$\lim_{n\to \infty }D(\mu ||\mu_{n})=0.$$

*Furthermore, $\lim_{n\to \infty }H\left(\mu_{n}\right)=H\left(\mu \right)$, if and only if,*

(10) $$\lim_{n\to \infty }\mu_{n}\left(A_{\mu_{n}}\backslash A_{\mu }\right)\cdot H\left(\mu_{n}\left(\cdot |A_{\mu_{n}}\backslash A_{\mu }\right)\right)=0\Leftrightarrow \lim_{n\to \infty }\sum_{x\in A_{\mu_{n}}\backslash A_{\mu }}f_{\mu_{n}}\left(x\right)\log\frac{1}{f_{\mu_{n}}\left(x\right)}=0,$$

*where μ(·|B) denotes the conditional probability of μ given the event B⊂X.,*

Lemma 1 tells us that to achieve entropy convergence (on top of the weak convergence), it is necessary and sufficient to ask for a vanishing expression (with *n*) of the entropy of $\mu_{n}$ restricted to the elements of the set $A_{\mu_{n}}\backslash A_{\mu }$. Two remarks about this result are: (1) The convergence in direct *I*-divergence does not imply the convergence of the entropy (concrete examples are presented in [24] (Section III) and [25]); (2) Under the assumption that μ∈F(X), μ is eventually absolutely continuous with respect to $\mu_{n}$, and the convergence in total variations is equivalent to the convergence in direct I-divergence.

This section concludes with the case when the support of μ is infinite and unknown, i.e., $\left|A_{\mu }\right|=\infty$. In this context, two results are highlighted:

**Lemma** **2.** *Ref. [31] (Theorem 4) Let us consider that μ∈H(X) and $\left\{\mu_{n}:n\geq 0\right\}\subset \mathrm{AC}\left(X|\mu \right)$. If $\mu_{n}\Rightarrow \mu$ and*

(11) $$M\equiv \underset{n\geq 1}{\sup}\underset{x\in A_{\mu }}{\sup}\frac{f_{\mu_{n}}\left(x\right)}{f_{\mu }\left(x\right)}<\infty ,$$

*then $\mu_{n}\in H\left(X\right)\cap H\left(X|\mu \right)$ for all n and it follows that*

$$\lim_{n\to \infty }D(\mu_{n}\left||\mu \right)=0\;\mathrm{and}\;\lim_{n\to \infty }H\left(\mu_{n}\right)=H\left(\mu \right).$$

Interpreting Lemma 2 we have that, to obtain the convergence of the entropy functional (without imposing a finite support assumption on μ), a uniform bounding condition (UBC) μ-almost everywhere was added in (11). By adding this UBC, the convergence on reverse I-divergence is also obtained as a byproduct. Finally, when $\mu \ll \mu_{n}$ for all *n*, the following result is considered:

**Lemma** **3.** *Ref. [25] (Theorem 3) Let μ∈H(X) and a sequence of measures $\left\{\mu_{n}:n\geq 1\right\}\subset H\left(X\right)$ such that $\mu \ll \mu_{n}$ for all n≥1. If $\mu_{n}\Rightarrow \mu$ and*

(12) $$\underset{n\geq 1}{\sup}\underset{x\in A_{\mu }}{\sup}\left|\log\frac{f_{\mu_{n}}\left(x\right)}{f_{\mu }\left(x\right)}\right|<\infty$$

*then, $\mu \in H(X|\mu_{n})$ for all n≥1, and*

$$\lim_{n\to \infty }D(\mu ||\mu_{n})=0.$$

*Furthermore, $\lim_{n\to \infty }H\left(\mu_{n}\right)=H\left(\mu \right)$, if and only if,*

(13) $$\lim_{n\to \infty }\sum_{x\in A_{\mu_{n}}\backslash A_{\mu }}f_{\mu_{n}}\left(x\right)\log\frac{1}{f_{\mu_{n}}\left(x\right)}=0.$$

This result shows the non-sufficiency of the convergence in direct I-divergence to achieve entropy convergence in the regime when $\mu \ll \mu_{n}$. In fact, Lemma 3 may be interpreted as an extension of Lemma 1 when the finite support assumption over μ is relaxed.

## 3. Shannon Entropy Estimation

Let μ be a probability in H(X), and let denote by $X_{1},X_{2},X_{3},\ldots$ the empirical process induced from i.i.d. realizations of a random variable driven by μ, i.e., $X_{i}∼\mu$, for all i≥0. Let $P_{\mu }$ denote the distribution of the empirical process in $\left(X^{\infty },B\left(X^{\infty }\right)\right)$ and $P_{\mu }^{n}$ denote the finite block distribution of $X^{n}\equiv \left(X_{1},\ldots ,X_{n}\right)$ in the product space $\left(X^{n},B\left(X^{n}\right)\right)$. Given a realization of $X_{1},X_{2},X_{3},\ldots ,X_{n}$, we can construct an histogram-based estimator like classical empirical probability given by:

(14) $${\hat{\mu }}_{n}\left(A\right)\equiv \frac{1}{n}\sum_{k=1}^{n}1_{A}\left(X_{k}\right),\;\forall A\subset X,$$

with pmf given by $f_{{\hat{\mu }}_{n}}\left(x\right)={\hat{\mu }}_{n}\left(\left\{x\right\}\right)$ for all x∈X. A natural estimator of the entropy is the plug-in estimate of ${\hat{\mu }}_{n}$ given by

(15) $$H\left({\hat{\mu }}_{n}\right)=-\sum_{x\in X}f_{{\hat{\mu }}_{n}}\left(x\right)\log f_{{\hat{\mu }}_{n}}\left(x\right),$$

which is a measurable function of $X_{1},\ldots ,X_{n}$ (this dependency on the data will be implicit for the rest of the paper).

For the rest of this Section and Section 4 and Section 5, the convergence results in Section 2.1 are used to derive strong consistency results for plug-in histogram-based estimators, like $H({\hat{\mu }}_{n})$ in (15), as well as finite length concentration inequalities to obtain almost-sure rates of convergence for the overall estimation error $\left|H\left({\hat{\mu }}_{n}\right)-H\left(\mu \right)\right|$.

### 3.1. Revisiting the Classical Plug-In Estimator for Finite and Unknown Supported Distributions

We start by analyzing the case when μ has a finite but unknown support. A consequence of the strong law of large numbers [42,43] is that ∀x∈X, $\lim_{n\to \infty }{\hat{\mu }}_{n}\left(\left\{x\right\}\right)=\mu \left(\left\{x\right\}\right)$, $P_{\mu }$-almost surely (a.s.), hence $\lim_{n\to \infty }V\left({\hat{\mu }}_{n},\mu \right)=0$, $P_{\mu }$-a.s. On the other hand, it is clear that $A_{{\hat{\mu }}_{n}}\subset A_{\mu }$ holds with probability one. Then Proposition 1 implies that

(16) $$\begin{array}{c} \lim_{n\to \infty }D({\hat{\mu }}_{n}\left||\mu \right)=0\;\mathrm{and}\;\lim_{n\to \infty }H\left({\hat{\mu }}_{n}\right)=H\left(\mu \right),\;P_{\mu }\text{-}a.s., \end{array}$$

i.e., ${\hat{\mu }}_{n}$ is a strongly consistent estimator of μ in reverse I-divergence and $H({\hat{\mu }}_{n})$ is a strongly consistent estimate of H(μ) distribution-free in F(X). Furthermore, the following can be stated:

**Theorem** **1.** *Let μ∈F(X) and let us consider ${\hat{\mu }}_{n}$ in (14). Then ${\hat{\mu }}_{n}\in H\left(X\right)\cap H\left(X|\mu \right)$, $P_{\mu }$-a.s and ∀n≥1, ∀ϵ>0,*

(17) $$\begin{array}{c} P_{\mu }^{n}\left(D({\hat{\mu }}_{n}||\mu )>\epsilon \right)\leq 2^{\left|A_{\mu }\right|+1}\cdot e^{-\frac{2m_{\mu }^{2}\cdot n\epsilon^{2}}{\log e^{2}}}, \end{array}$$

(18) $$\begin{array}{c} P_{\mu }^{n}\left(\left|H\left({\hat{\mu }}_{n}\right)-H\left(\mu \right)\right|>\epsilon \right)\leq 2^{\left|A_{\mu }\right|+1}\cdot e^{-\frac{2n\epsilon^{2}}{{\left(M_{\mu }+\frac{\log e}{m_{\mu }}\right)}^{2}}}. \end{array}$$

*Moreover, $D(\mu ||{\hat{\mu }}_{n})$ is eventually finite with probability one and ∀ϵ>0, and for any n≥1,*

(19) $$\begin{array}{cc} & P_{\mu }^{n}\left(D(\mu ||{\hat{\mu }}_{n})>\epsilon \right)\leq 2^{\left|A_{\mu }\right|+1}\cdot \left[e^{-\frac{2n\epsilon^{2}}{\log e^{2}\cdot {\left(1/m_{u}+1\right)}^{2}}}+e^{-nm_{\mu }^{2}}\right]. \end{array}$$

This result implies that for any τ∈(0,1/2) and μ∈F(X), $\left|H\left({\hat{\mu }}_{n}\right)-H\left(\mu \right)\right|$, $D({\hat{\mu }}_{n}||\mu )$, and $D(\mu ||{\hat{\mu }}_{n})$ goes to zero as $o(n^{-\tau })$ $P_{\mu }$-a.s. Furthermore, $E_{P_{\mu }^{n}}\left(\left|H\left({\hat{\mu }}_{n}\right)-H\left(\mu \right)\right|\right)$ and $E_{P_{\mu }^{n}}(D({\hat{\mu }}_{n}\left||\mu )\right)$ behave like $O(1/\sqrt{n})$ for all μ∈F(X) from (30) in Section 7, which is the optimal rate of convergence of the finite alphabet scenario. As a corollary of (18), it is possible to derive intervals of confidence for the estimation error $\left|H(\left({\hat{\mu }}_{n}\right)-H\left(\mu_{n}\right)\right|$: for all δ>0 and n≥1,

(20) $$\begin{array}{c} P_{\mu }\left(\left|H(\left({\hat{\mu }}_{n}\right)-H\left(\mu_{n}\right)\right|\leq \left(M_{\mu }+\log e/m_{\mu }\right)\sqrt{\frac{1}{2n}\ln\frac{2^{\left|A_{\mu }\right|+1}}{\delta }}\right)\geq 1-\delta . \end{array}$$

This confidence interval behaves like $O(1/\sqrt{n})$ as a function of *n*, and like $O(\sqrt{\ln1/\delta })$ as a function of δ, which are the same optimal asymptotic trends that can be obtained for $V(\mu ,{\hat{\mu }}_{n})$ in (30).

Finally, we observe that $A_{{\hat{\mu }}_{n}}\subset A_{\mu }$ $P_{\mu }^{n}$-a.s. where for any n≥1, $P_{\mu }^{n}\left(A_{{\hat{\mu }}_{n}}\neq A_{\mu }\right)>0$ implying that $E_{P_{\mu }^{n}}\left(D(\mu |\right|{\hat{\mu }}_{n}))=\infty$ for all finite *n*. Then even in the finite and unknown supported scenario, ${\hat{\mu }}_{n}$ is not consistent in expected direct I-divergence, which is congruent with the result in [29,44]. Besides this negative result, strong consistency in direct *I*-divergence can be obtained from (19), in the sense that $\lim_{n\to \infty }D(\mu ||{\hat{\mu }}_{n})=0$, $P_{\mu }$-a.s.

### 3.2. A Simplified Version of the Barron Estimator for Finite Supported Probabilities

It is well-understood that consistency in expected direct I-divergence is of critical importance for the construction of a lossless universal source coding scheme [2,23,29,44,45,46,47,48]. Here, we explore an estimator that achieves this learning objective, in addition to entropy estimation. For that, let μ∈F(X) and let assume v∈F(X) such that μ≪v. Barron et al. [29] proposed a modified version of the empirical measure in (14) to estimate μ from i.i.d. realizations, adopting a mixture estimate of the form

(21) $$\begin{array}{c} {\tilde{\mu }}_{n}\left(B\right)=\left(1-a_{n}\right)\cdot {\hat{\mu }}_{n}\left(B\right)+a_{n}\cdot v\left(B\right), \end{array}$$

for all B⊂X, and with ${\left(a_{n}\right)}_{n\in N}$ a sequence of real numbers in (0,1). Note that $A_{{\tilde{\mu }}_{n}}=A_{v}$ then $\mu \ll {\tilde{\mu }}_{n}$ for all *n* and from the finite support assumption $H({\tilde{\mu }}_{n})<\infty$ and $D(\mu ||{\tilde{\mu }}_{n})<\infty$, $P_{\mu }$-a.s.. The following result derives from the convergence result in Lemma 1.

**Theorem** **2.** *Let v∈F(X), μ≪v and let us consider ${\tilde{\mu }}_{n}$ in (21) induced from i.i.d. realizations of μ.*

*(i)*  If $\left(a_{n}\right)$ is o(1), then $\lim_{n\to \infty }H\left({\tilde{\mu }}_{n}\right)=H\left(\mu \right)$, $\lim_{n\to \infty }D(\mu ||{\tilde{\mu }}_{n})=0$, $P_{\mu }$-a.s., and $\lim_{n\to \infty }E_{P_{\mu }}\left(D(\mu |\right|{\tilde{\mu }}_{n}))=0$.
*(ii)*  Furthermore, if $\left(a_{n}\right)$ is $O(n^{-p})$ with p>2, then for all τ∈(0,1/2), $\left|H\left({\tilde{\mu }}_{n}\right)-H\left(\mu \right)\right|$ and $D(\mu ||{\tilde{\mu }}_{n})$ are $o(n^{-\tau })$$P_{\mu }$-a.s, and $E_{P_{\mu }}\left(\left|H\left({\tilde{\mu }}_{n}\right)-H\left(\mu \right)\right|\right)$ and $E_{P_{\mu }}\left(D(\mu |\right|{\tilde{\mu }}_{n}))$ are $O(1/\sqrt{n})$.

Using this approach, we achieve estimation of the true distribution in expected information divergence as well as strong consistency for entropy estimation as intended. In addition, optimal rates of convergence are obtained under the finite support assumption on μ.

## 4. The Barron-Györfi-van der Meulen Estimator

The celebrated Barron estimator was proposed by Barron, Györfi and van der Meulen [29] in the context of an abstract and continuous measurable space. It is designed as a variation of the classical histogram-based scheme to achieve a consistent estimation of the distribution in direct I-divergence [29] (Theorem 2). Here, the Barron estimator is revisited in the countable alphabet scenario, with the objective of estimating the Shannon entropy consistently, which, to the best of our knowledge, has not been previously addressed in literature. For that purpose, the convergence result in Lemma 3 will be used as a key result.

Let v∈P(X) be of infinite support (i.e., $m_{v}=\inf_{x\in A_{v}}v\left(\left\{x\right\}\right)=0$). We want to construct a strongly consistent estimate of the entropy restricted to the collection of probabilities in H(X|v). For that, let us consider a sequence ${\left(h_{n}\right)}_{n\geq 0}$ with values in (0,1) and let us denote by $\pi_{n}=\left\{A_{n,1},A_{n,2},\ldots ,A_{n,m_{n}}\right\}$ the finite partition of X with maximal cardinality satisfying that

(22) $$v\left(A_{n,i}\right)\geq h_{n},\;\forall i\in \left\{1,\ldots ,m_{n}\right\}.$$

Note that $m_{n}=\left|\pi_{n}\right|\leq 1/h_{n}$ for all n≥1, and because of the fact that $\inf_{x\in A_{v}}v\left(\left\{x\right\}\right)=0$ it is simple to verify that if $\left(h_{n}\right)$ is o(1) then $\lim_{n\to \infty }m_{n}=\infty$. $\pi_{n}$ offers an approximated statistically equivalent partition of X with respect to the reference measure *v*. In this context, given $X_{1},\ldots ,X_{n}$, i.i.d. realizations of μ∈H(X|v), the idea proposed by Barron et al. [29] is to estimate the RN derivative $\frac{d\mu }{dv}\left(x\right)$ by the following histogram-based construction:

(23) $$\frac{d\mu_{n}^{*}}{dv}\left(x\right)=\left(1-a_{n}\right)\cdot \frac{{\hat{\mu }}_{n}\left(A_{n}\left(x\right)\right)}{v(A_{n}\left(x\right))}+a_{n},\;\forall x\in A_{v},$$

where $a_{n}$ is a real number in (0,1), $A_{n}\left(x\right)$ denotes the cell in $\pi_{n}$ that contains the point *x*, and ${\hat{\mu }}_{n}$ is the empirical measure in (14). Note that

$$f_{\mu_{n}^{*}}\left(x\right)=\frac{d\mu_{n}^{*}}{d\lambda }\left(x\right)=f_{v}\left(x\right)\cdot \left[\left(1-a_{n}\right)\cdot \frac{{\hat{\mu }}_{n}\left(A_{n}\left(x\right)\right)}{v(A_{n}\left(x\right))}+a_{n}\right],$$

∀x∈X, and, consequently, ∀B⊂X

(24) $$\mu_{n}^{*}\left(B\right)=\left(1-a_{n}\right)\sum_{i=1}^{m_{n}}{\hat{\mu }}_{n}\left(A_{n,i}\right)\cdot \frac{v(B\cap A_{n,i})}{v(A_{n,i})}+a_{n}v\left(B\right).$$

By construction $A_{\mu }\subset A_{v}\subset A_{\mu_{n}^{*}}$ and, consequently, $\mu \ll \mu_{n}^{*}$ for all n≥1. The next result shows sufficient conditions on the sequences $\left(a_{n}\right)$ and $\left(h_{n}\right)$ to guarantee a strongly consistent estimation of the entropy H(μ) and of μ in direct *I*-divergence, distribution free in H(X|v). The proof is based on verifying that the sufficient conditions of Lemma 3 are satisfied $P_{\mu }$-a.s.

**Theorem** **3.** *Let v be in P(X)∩H(X) with infinite support, and let us consider μ in H(X|v). If we have that:*

*(i)*  $\left(a_{n}\right)$ is o(1) and $\left(h_{n}\right)$ is o(1),
*(ii)*  ∃τ∈(0,1/2), such that the sequence $\left(\frac{1}{a_{n}\cdot h_{n}}\right)$ is $o(n^{\tau })$,

then $\mu \in H\left(X\right)\cap H\left(X|\mu_{n}^{*}\right)$ for all n≥1 and

(25) $$\lim_{n\to \infty }H\left(\mu_{n}^{*}\right)=H\left(\mu \right)\;\mathrm{and}\;\lim_{n\to \infty }D(\mu ||\mu_{n}^{*})=0,\;P_{\mu }\text{-}a.s..$$

This result shows an admisible regime of design parameters and its scaling with the number of samples that guarantees that the Barron plug-in entropy estimator is strongly consistent in H(X|v). As a byproduct, we obtain that the distribution μ is estimated consistently in direct information divergence.

The Barron estimator [29] was originally proposed in the context of distributions defined in an abstract measurable space. Then if we restrict [29] (Theorem 2) to the countable alphabet case, the following result is obtained:

**Corollary** **1.** *Ref. [29] (Theorem 2) Let us consider v∈P(X) and μ∈H(X|v). If $\left(a_{n}\right)$ is o(1), $\left(h_{n}\right)$ is o(1) and $\lim\sup_{n\to \infty }\frac{1}{na_{n}h_{n}}\leq 1$ then*

$$\lim_{n\to \infty }D(\mu ||\mu_{n}^{*})=0,\;P_{\mu }\text{-}a.s.$$

When the only objective is the estimation of distributions consistently in direct I-divergence, Corollary 1 should be considered to be a better result than Theorem 3 (Corollary 1 offers weaker conditions than Theorem 3 in particular condition (ii)). The proof of Theorem 3 is based on verifying the sufficient conditions of Lemma 3, where the objective is to achieve the convergence of the entropy, and as a consequence, the convergence in direct I-divergence. Therefore, we can say that the stronger conditions of Theorem 3 are needed when the objective is entropy estimation. This is justified from the observation that convergence in direct I-divergence does not imply entropy convergence in ∞-alphabets, as is discussed in Section 2.1 (see, Lemmas 1 and 3).

## 5. A Data-Driven Histogram-Based Estimator

Data-driven partitions offer a better approximation to the data distribution in the sample space than conventional non-adaptive histogram-based approaches [34,49]. They have the capacity to improve the approximation quality of histogram-based learning schemes, which translates in better performance in different non-parametric learning settings [34,35,36,50,51]. One of the basic design principles of this approach is to partition or select a sub-set of elements of X in a data-dependent way to preserve a critical number of samples per cell. In our problem, this last condition proves to be crucial to derive bounds for the estimation and approximation errors. Finally, these expressions will be used to propose design solutions that offer an optimal balance between estimation and approximation errors (Theorems 5 and 6).

Given $X_{1},\ldots ,X_{n}$ i.i.d. realizations driven by μ∈H(X) and ϵ>0, let us define the data-driven set

(26) $$\Gamma_{\epsilon }\equiv \left\{x\in X:{\hat{\mu }}_{n}\left(\left\{x\right\}\right)\geq \epsilon \right\},$$

and $\phi_{\epsilon }\equiv \Gamma_{\epsilon }^{c}$. Let $\Pi_{\epsilon }\equiv \left\{\left\{x\right\}:x\in \Gamma_{\epsilon }\right\}\cup \left\{\phi_{\epsilon }\right\}\subset 2^{X}$ be a data-driven partition with maximal resolution in $\Gamma_{\epsilon }$, and $\sigma_{\epsilon }\equiv \sigma \left(\Pi_{\epsilon }\right)$ be the smallest sigma field that contains $\Pi_{\epsilon }$ (as $\Pi_{\epsilon }$ is a finite partition, $\sigma_{\epsilon }$ is the collection of sets that are union of elements of $\Pi_{\epsilon }$). We propose the conditional empirical probability restricted to $\Gamma_{\epsilon }$ by:

(27) $${\hat{\mu }}_{n,\epsilon }\equiv {\hat{\mu }}_{n}\left(\cdot |\Gamma_{\epsilon }\right).$$

By construction, it follows that $A_{{\hat{\mu }}_{n,\epsilon }}=\Gamma_{\epsilon }\subset A_{\mu }$, $P_{\mu }$-a.s. and this implies that ${\hat{\mu }}_{n,\epsilon }\ll \mu$ for all n≥1. Furthermore, $\left|\Gamma_{\epsilon }\right|\leq \frac{1}{\epsilon }$ and, importantly in the context of the entropy functional, it follows that

(28) $$m_{{\hat{\mu }}_{n}}^{\epsilon }\equiv \underset{x\in \Gamma_{\epsilon }}{\inf}{\hat{\mu }}_{n}\left(\left\{x\right\}\right)\geq \epsilon .$$

The next result establishes a mild sufficient condition on $\left(\epsilon_{n}\right)$ for which $H({\hat{\mu }}_{n,\epsilon_{n}})$ is strongly consistent distribution-free in H(X). Considering that we are in the regime where ${\hat{\mu }}_{n,\epsilon_{n}}\ll \mu$, $P_{\mu }$-a.s., the proof of this result uses the convergence result in Lemma 2 as a central result.

**Theorem** **4.** *If $\left(\epsilon_{n}\right)$ is $O(n^{-\tau })$ with τ∈(0,1), then for all μ∈H(X)*

$$\lim_{n\to \infty }H\left({\hat{\mu }}_{n,\epsilon_{n}}\right)=H\left(\mu \right),\;P_{\mu }\text{-}a.s.$$

Complementing Theorem 4, the next result offers almost-sure rates of converge for a family of distributions with a power tail bounded condition (TBC). In particular, the family of distributions studied in [30] (Theorem 7) are considered.

**Theorem** **5.** Let us assume that for some p>1 there are two constants $0<k_{0}\leq k_{1}$ and N>0 such that $k_{0}\cdot x^{-p}\leq \mu \left(\left\{x\right\}\right)\leq k_{1}x^{-p}$ for all x≥N. If we consider that $\left(\epsilon_{n}\right)\approx \left(n^{-\tau^{*}}\right)$ for $\tau^{*}=\frac{1}{2+1/p}$, then

$$\left|H\left(\mu \right)-H\left({\hat{\mu }}_{n,\epsilon_{n}}\right)\right|\;\mathrm{is}\;O\left(n^{-\frac{1-1/p}{2+1/p}}\right),\;P_{\mu }\text{-}a.s.$$

This result shows that under the mentioned *p*-power TBC on $f_{\mu }\left(\cdot \right)$, the plug-in estimator $H({\hat{\mu }}_{n,\epsilon_{n}})$ can achieve a rate of convergence to the true limit that is $O(n^{-\frac{1-1/p}{2+1/p}})$ with probability one. For the derivation of this result, the approximation sequence $\left(\epsilon_{n}\right)$ is defined as a function of *p* (adapted to the problem) by finding an optimal tradeoff between estimation and approximation errors while performing a finite length (non-asymptotic) analysis of the expression $\left|H\left(\mu \right)-H\left({\hat{\mu }}_{n,\epsilon_{n}}\right)\right|$ (the details of this analysis are presented in Section 7).

It is insightful to look at two extreme regimes of this result: *p* approaching 1, in which the rate is arbitrarily slow (approaching a non-decaying behavior); and p→∞, where $\left|H\left(\mu \right)-H\left({\hat{\mu }}_{n,\epsilon_{n}}\right)\right|$ is $O(n^{-q})$ for all q∈(0,1/2) $P_{\mu }$-a.s.. This last power decaying range q∈(0,1/2) matches what is achieved for the finite alphabet scenario (for instance in Theorem 1, Equation (18)), which is known to be the optimal rate for finite alphabets.

Extending Theorem 5, the following result addresses the more constrained case of distributions with an exponential TBC.

**Theorem** **6.** *Let us consider α>0 and let us assume that there are $k_{0},k_{1}$ with $0<k_{0}\leq k_{1}$ and N>0 such that $k_{0}\cdot e^{-\alpha x}\leq \mu \left(\left\{x\right\}\right)\leq k_{1}\cdot e^{-\alpha x}$ for all x≥N. If we consider $\left(\epsilon_{n}\right)\approx \left(n^{-\tau }\right)$ with τ∈(0,1/2), then*

$$\left|H\left(\mu \right)-H\left({\hat{\mu }}_{n,\epsilon_{n}}\right)\right|\;\mathrm{is}\;O\left(n^{-\tau }\log n\right),\;P_{\mu }\text{-}a.s.$$

Under this stringent TBC on $f_{\mu }\left(\cdot \right)$, it is observed that $\left|H\left(\mu \right)-H\left({\hat{\mu }}_{n,\epsilon_{n}}\right)\right|\;\mathrm{is}\;o\left(n^{-q}\right)$ $P_{\mu }\text{-}a.s.$, for any arbitrary q∈(0,1/2), by selecting $\left(\epsilon_{n}\right)\approx \left(n^{-\tau }\right)$ with q<τ<1/2. This last condition on τ is universal over α>0. Remarkably, for any distribution with this exponential TBC, we can approximate (arbitrarily closely) the optimal almost-sure rate of convergence achieved for the finite alphabet problem.

Finally, the finite and unknown supported scenario is revisited, where it is shown that the data-driven estimator exhibits the optimal almost sure convergence rate of the classical plug-in entropy estimator presented in Section 3.1.

**Theorem** **7.** *Let us assume that μ∈F(X) and $\left(\epsilon_{n}\right)$ being o(1). Then for all ϵ>0 there is N>0 such that ∀n≥N*

(29) $$P_{\mu }^{n}\left(\left|H\left({\hat{\mu }}_{n,\epsilon_{n}}\right)-H\left(\mu \right)\right|>\epsilon \right)\leq 2^{\left|A_{\mu }\right|+1}\cdot \left[e^{-\frac{2n\epsilon^{2}}{{\left(M_{\mu }+\frac{\log e}{m_{\mu }}\right)}^{2}}}+e^{-\frac{nm_{\mu }^{2}}{4}}\right].$$

The proof of this result reduces to verify that ${\hat{\mu }}_{n,\epsilon_{n}}$ detects $A_{\mu }$ almost-surely when *n* goes to infinity and from this, it follows that $H({\hat{\mu }}_{n,\epsilon_{n}})$ eventually matches the optimal almost sure performance of $H({\hat{\mu }}_{n})$ under the key assumption that μ∈F(X). Finally, the concentration bound in (29) implies that $\left|H\left({\hat{\mu }}_{n,\epsilon_{n}}\right)-H\left(\mu \right)\right|$ is $o(n^{-q})$ almost surely for all q∈(0,1/2) as long as $\epsilon_{n}\to 0$ with *n*.

## 6. Discussion of the Results and Final Remarks

This work shows that entropy convergence results are instrumental to derive new (strongly consistent) estimation results for the Shannon entropy in ∞-alphabets and, as a byproduct, distribution estimators that are strongly consistent in direct and reverse I-divergence. Adopting a set of sufficient conditions for entropy convergence in the context of four plug-in histogram-based schemes, this work shows concrete design conditions where strong consistency for entropy estimation in ∞-alphabets can be obtained (Theorems 2–4). In addition, the relevant case where the target distribution has a finite but unknown support is explored, deriving almost sure rates of convergence results for the overall estimation error (Theorems 1 and 7) that match the optimal asymptotic rate that can be obtained in the finite alphabet version of the problem (i.e., the finite and known supported case).

As the main context of application, this work focuses on the case of a data-driven plug-in estimator that restricts the support where the distribution is estimated. The idea is to have design parameters that control estimation-error effects and to find an adequate balance between these two learning errors. Adopting the entropy convergence result in Lemma 2, it is shown that this data-driven scheme offers the same universal estimation attributes than the classical plug-in estimate under some mild conditions on its threshold design parameter (Theorem 4). In addition, by addressing the technical task of deriving concrete closed-form expressions for the estimation and approximation errors in this learning context a solution is presented where almost-sure rates of convergence of the overall estimation error are obtained over a family of distributions with some concrete tail bounded conditions (Theorems 5 and 6). These results show the capacity that data-driven frameworks offer for adapting aspects of their learning scheme to the complexity of the entropy estimation task in ∞-alphabets.

Concerning the classical plug-in estimator presented in Section 3.1, it is important to mention that the work of Antos et al. [30] shows that $\lim_{n\to \infty }H\left({\hat{\mu }}_{n}\right)=H\left(\mu \right)$ happens almost surely and distribution-free and, furthermore, it provides rates of convergence for families with specific tail-bounded conditions [30] (Theorem 7). Theorem 1 focuses on the case when μ∈F(X), where new finite-length deviation inequalities and confidence intervals are derived. From that perspective, Theorem 1 complements the results presented in [30] in the non-explored scenario when μ∈F(X). It is also important to mention two results by Ho and Yeung [26] (Theorems 11 and 12) for the plug-in estimator in (15). They derived bounds for $P_{\mu }^{n}\left(\left|H\left({\hat{\mu }}_{n}\right)-H\left(\mu \right)\right|\geq \epsilon \right)$ and determined confidence intervals under a finite and known support restriction on μ. In contrast, Theorem 1 resolves the case for a finite and unknown supported distribution, which is declared to be a challenging problem from the arguments presented in [26] (Theorem 13) concerning the discontinuity of the entropy.

## 7. Proof of the Main Results

**Proof of Theorem** **1.** Let μ be in F(X), then $\left|A_{\mu }\right|\leq k$ for some k>1. From Hoeffding’s inequality [28], ∀n≥1, and for any ϵ>0

(30) $$\begin{array}{c} P_{\mu }^{n}\left(V({\hat{\mu }}_{n},\mu )>\epsilon \right)\leq 2^{k+1}\cdot e^{-2n\epsilon^{2}}\;\mathrm{and}\;E_{P_{\mu }^{n}}\left(V\left({\hat{\mu }}_{n},\mu \right)\right)\leq 2\sqrt{\frac{\left(k+1\right)\log2}{n}}. \end{array}$$

Considering that ${\hat{\mu }}_{n}\ll \mu$ $P_{\mu }$-a.s, we can use Proposition 1 to obtain that

(31) $$\begin{array}{c} D({\hat{\mu }}_{n}\left||\mu \right)\leq \frac{\log e}{m_{\mu }}\cdot V\left({\hat{\mu }}_{n},\mu \right),\;\mathrm{and}\;\left|H(\left({\hat{\mu }}_{n}\right)-H\left(\mu_{n}\right)\right|\leq \left[M_{\mu }+\frac{\log e}{m_{\mu }}\right]\cdot V\left({\hat{\mu }}_{n},\mu \right). \end{array}$$

Hence, (17) and (18) derive from (30).

For the direct I-divergence, let us consider a sequence ${\left(x_{i}\right)}_{i\geq 1}$ and the following function (a stopping time):

(32) $$\begin{array}{c} T_{o}\left(x_{1},x_{2},\ldots \right)\equiv \inf\left\{n\geq 1:A_{{\hat{\mu }}_{n}\left(x^{n}\right)}=A_{\mu }\right\}. \end{array}$$

$T_{o}\left(x_{1},x_{2},\ldots \right)$ is the point where the support of ${\hat{\mu }}_{n}\left(x^{n}\right)$ is equal to $A_{\mu }$ and, consequently, the direct I-divergence is finite (since μ∈F(X)). In fact, by the uniform convergence of ${\hat{\mu }}_{n}$ to $\mu_{n}$ ($P_{\mu }$-a.s.) and the finite support assumption of μ, it is simple to verify that $P_{\mu }\left(T_{o}\left(X_{1},X_{2},\ldots \right)<\infty \right)=1$. Let us define the event:

(33) $$\begin{array}{c} B_{n}\equiv \left\{x_{1},x_{2},\ldots :T_{o}\left(x_{1},x_{2},\ldots \right)\leq n\right\}\subset X^{N}, \end{array}$$

i.e., the collection of sequences in $X^{N}$ where at time *n*, $A_{{\hat{\mu }}_{n}}=A_{\mu }$ and, consequently, $D(\mu ||{\hat{\mu }}_{n})<\infty$. Restricted to this set

(34) $$\begin{array}{cc} D(\mu ||{\hat{\mu }}_{n}) & \leq \sum_{x\in A_{{\hat{\mu }}_{n}\left|\right|\mu }}f_{{\hat{\mu }}_{n}}\left(x\right)\log\frac{f_{{\hat{\mu }}_{n}}\left(x\right)}{f_{\mu }\left(x\right)}+\sum_{x\in A_{\mu }\backslash A_{{\hat{\mu }}_{n}\left|\right|\mu }}f_{{\hat{\mu }}_{n}}\left(x\right)\log\frac{f_{\mu }\left(x\right)}{f_{{\hat{\mu }}_{n}}\left(x\right)}\\ & \leq \log e\cdot \sum_{x\in A_{{\hat{\mu }}_{n}\left|\right|\mu }}f_{{\hat{\mu }}_{n}}\left(x\right)\cdot \left(\frac{f_{{\hat{\mu }}_{n}}\left(x\right)}{f_{\mu }\left(x\right)}-1\right) \end{array}$$

(35) $$\begin{array}{c} +\log e\cdot \left[\mu \left(A_{\mu }\backslash A_{{\hat{\mu }}_{n}\left|\right|\mu }\right)-{\hat{\mu }}_{n}\left(\left(A_{\mu }\backslash A_{{\hat{\mu }}_{n}\left|\right|\mu }\right)\right)\right] \end{array}$$

(36) $$\begin{array}{c} \leq \log e\cdot \left(1/m_{u}+1\right)V\left(\mu ,{\hat{\mu }}_{n}\right), \end{array}$$

where in the first inequality $A_{{\hat{\mu }}_{n}\left|\right|\mu }\equiv \left\{x\in A_{{\hat{\mu }}_{n}}:f_{{\hat{\mu }}_{n}}\left(x\right)>f_{\mu }\left(x\right)\right\}$, and the last is obtained by the definition of the total variational distance. In addition, let us define the ϵ-deviation set $A_{n}^{\epsilon }\equiv \left\{x_{1},x_{2},\ldots :D(\mu ||{\hat{\mu }}_{n}\left(x^{n}\right))>\epsilon \right\}\subset X^{N}$. Then by additivity and monotonicity of $P_{\mu }$, we have that

(37) $$\begin{array}{c} P_{\mu }\left(A_{n}^{\epsilon }\right)\leq P_{\mu }\left(A_{n}^{\epsilon }\cap B_{n}\right)+P_{\mu }\left(B_{n}^{c}\right). \end{array}$$

By definition of $B_{n}$, (36) and (30) it follows that

(38) $$\begin{array}{cc} P_{\mu }\left(A_{n}^{\epsilon }\cap B_{n}\right) & \leq P_{\mu }\left(V(\mu |\right|{\hat{\mu }}_{n})\log e\cdot \left(1/m_{u}+1\right)>\epsilon )\\ & \leq 2^{\left|A_{\mu }\right|+1}\cdot e^{-\frac{2n\epsilon^{2}}{\log e^{2}\cdot {\left(1/m_{u}+1\right)}^{2}}}. \end{array}$$

On the other hand, $\forall \epsilon_{o}\in \left(0,m_{\mu }\right)$ if $V\left(\mu ,{\hat{\mu }}_{n}\right)\leq \epsilon_{o}$ then $T_{o}\leq n$. Consequently $B_{n}^{c}\subset \left\{x_{1},x_{2},\ldots :V\left(\mu ,{\hat{\mu }}_{n}\left(x^{n}\right)\right)>\epsilon_{o}\right\}$, and again from (30)

(39) $$\begin{array}{cc} P_{\mu }\left(B_{n}^{c}\right) & \leq 2^{\left|A_{\mu }\right|+1}\cdot e^{-2n\epsilon_{o}^{2}}, \end{array}$$

for all n≥1 and $\forall \epsilon_{o}\in \left(0,m_{\mu }\right)$. Integrating the results in (38) and (39) and considering $\epsilon_{0}=m_{\mu }/\sqrt{2}$ suffice to show the bound in (19). ☐

**Proof of Theorem** **2.** As $\left(a_{n}\right)$ is o(1), it is simple to verify that $\lim_{n\to \infty }V\left({\tilde{\mu }}_{n},\mu \right)=0$, $P_{\mu }$-a.s. Also note that the support disagreement between ${\tilde{\mu }}_{n}$ and μ is bounded by the hypothesis, then

(40) $$\begin{array}{c} \lim_{n\to \infty }{\tilde{\mu }}_{n}\left(A_{\mu_{n}}\backslash A_{\mu }\right)\cdot \log\left|A_{{\tilde{\mu }}_{n}}\backslash A_{\mu }\right|\leq \lim_{n\to \infty }{\tilde{\mu }}_{n}\left(A_{\mu_{n}}\backslash A_{\mu }\right)\cdot \log\left|A_{v}\right|=0,\;P_{\mu }\text{-}a.s. \end{array}$$

Therefore from Lemma 1, we have the strong consistency of $H({\tilde{\mu }}_{n})$ and the almost sure convergence of $D(\mu ||{\tilde{\mu }}_{n})$ to zero. Note that $D(\mu ||{\tilde{\mu }}_{n})$ is uniformly upper bounded by $\log e\cdot \left(1/m_{\mu }+1\right)V\left(\mu ,{\tilde{\mu }}_{n}\right)$ (see (36) in the proof of Theorem 1). Then the convergence in probability of $D(\mu ||{\tilde{\mu }}_{n})$ implies the convergence of its mean [42], which concludes the proof of the first part.

Concerning rates of convergence, we use the following:

(41) $$\begin{array}{cc} H\left(\mu \right)-H\left({\tilde{\mu }}_{n}\right) & =\sum_{x\in A_{\mu }\cap A_{{\tilde{\mu }}_{n}}}\left[f_{{\tilde{\mu }}_{n}}\left(x\right)-f_{\mu }\left(x\right)\right]\log f_{\mu }\left(x\right)+\sum_{x\in A_{\mu }\cap A_{{\tilde{\mu }}_{n}}}f_{{\tilde{\mu }}_{n}}\left(x\right)\log\frac{f_{{\tilde{\mu }}_{n}}\left(x\right)}{f_{\mu }\left(x\right)}\\ & -\sum_{x\in A_{{\tilde{\mu }}_{n}}\backslash A_{\mu }}f_{{\tilde{\mu }}_{n}}\left(x\right)\log\frac{1}{f_{{\tilde{\mu }}_{n}}\left(x\right)}. \end{array}$$

The absolute value of the first term in the right hand side (RHS) of (41) is bounded by $M_{\mu }\cdot V\left({\tilde{\mu }}_{n},\mu \right)$ and the second term is bounded by $\log e/m_{\mu }\cdot V\left({\tilde{\mu }}_{n},\mu \right)$, from the assumption that μ∈F(X). For the last term, note that $f_{{\tilde{\mu }}_{n}}\left(x\right)=a_{n}\cdot v\left(\left\{x\right\}\right)$ for all $x\in A_{{\tilde{\mu }}_{n}}\backslash A_{\mu }$ and that $A_{{\tilde{\mu }}_{n}}=A_{v}$, then

$$0\leq \sum_{x\in A_{{\tilde{\mu }}_{n}}\backslash A_{\mu }}f_{{\tilde{\mu }}_{n}}\left(x\right)\log\frac{1}{f_{{\tilde{\mu }}_{n}}\left(x\right)}\leq a_{n}\cdot \left(H\left(v\right)+\log\frac{1}{a_{n}}\cdot v\left(A_{v}\backslash A_{\mu }\right)\right).$$

On the other hand,

$$\begin{array}{cc} V({\tilde{\mu }}_{n},\mu ) & =\frac{1}{2}\sum_{x\in A_{\mu }}\left|\left(1-a_{n}\right){\hat{\mu }}_{n}\left(\left\{x\right\}\right)+a_{n}v\left(\left\{x\right\}\right)-\mu \left(\left\{x\right\}\right)\right|+\sum_{x\in A_{v}\backslash A_{\mu }}a_{n}v\left(\left\{x\right\}\right).\\ & \leq \left(1-a_{n}\right)\cdot V\left({\hat{\mu }}_{n},\mu \right)+a_{n}. \end{array}$$

Integrating these bounds in (41),

(42) $$\begin{array}{cc} \left|H\left(\mu \right)-H\left({\tilde{\mu }}_{n}\right)\right| & \leq \left(M_{\mu }+\log e/m_{\mu }\right)\cdot \left(\left(1-a_{n}\right)\cdot V\left({\hat{\mu }}_{n},\mu \right)+a_{n}\right)+a_{n}\cdot H\left(v\right)+a_{n}\cdot \log\frac{1}{a_{n}}\\ & =K_{1}\cdot V\left({\hat{\mu }}_{n},\mu \right)+K_{2}\cdot a_{n}+a_{n}\cdot \log\frac{1}{a_{n}}, \end{array}$$

for constants $K_{1}>0$ and $K_{2}>0$ function of μ and *v*.

Under the assumption that μ∈F(X), the Hoeffding’s inequality [28,52] tells us that $P_{\mu }\left(V\left({\hat{\mu }}_{n},\mu \right)>\epsilon \right)\leq C_{1}\cdot e^{-C_{2}n\epsilon^{2}}$ (for some distribution free constants $C_{1}>0$ and $C_{2}>0$). From this inequality, $V({\hat{\mu }}_{n},\mu )$ goes to zero as $o(n^{-\tau })$ $P_{\mu }$-a.s. ∀τ∈(0,1/2) and $E_{P_{\mu }}\left(V\left({\hat{\mu }}_{n},\mu \right)\right)$ is $O(1/\sqrt{n})$. On the other hand, under the assumption in ii) $\left(K_{2}\cdot a_{n}+a_{n}\cdot \log\frac{1}{a_{n}}\right)$ is $O(1/\sqrt{n})$, which from (42) proves the rate of convergence results for $\left|H\left(\mu \right)-H\left({\tilde{\mu }}_{n}\right)\right|$.

Considering the direct I-divergence, $D(\mu ||{\tilde{\mu }}_{n})\leq \log e\cdot \sum_{x\in A_{\mu }}f_{\mu }\left(x\right)\left|\frac{f_{\mu }\left(x\right)}{f_{{\tilde{\mu }}_{n}}\left(x\right)}-1\right|\leq \frac{\log e}{m_{{\tilde{\mu }}_{n}}}\cdot V\left({\tilde{\mu }}_{n},\mu \right)$. Then the uniform convergence of ${\tilde{\mu }}_{n}\left(\left\{x\right\}\right)$ to $\mu (\left\{x\right\})$ $P_{\mu }$-a.s. in $A_{\mu }$ and the fact that $\left|A_{\mu }\right|<\infty$ imply that for an arbitrary small ϵ>0 (in particular smaller than $m_{\mu }$)

(43) $$\begin{array}{cc} \lim_{n⟶\infty }D(\mu ||{\tilde{\mu }}_{n}) & \leq \frac{\log e}{m_{\mu }-\epsilon }\cdot \lim_{n⟶\infty }V\left({\tilde{\mu }}_{n},\mu \right),\;P_{\mu }\text{-}a.s.. \end{array}$$

(43) suffices to obtain the convergence result for the I-divergence. ☐

**Proof of Theorem** **3.** Let us define the oracle Barron measure ${\tilde{\mu }}_{n}$ by:

(44) $$f_{{\tilde{\mu }}_{n}}\left(x\right)=\frac{d{\tilde{\mu }}_{n}}{d\lambda }\left(x\right)=f_{v}\left(x\right)\left[\left(1-a_{n}\right)\cdot \frac{\mu (A_{n}\left(x\right))}{v(A_{n}\left(x\right))}+a_{n}\right],$$

where we consider the true probability instead of its empirical version in (23). Then, the following convergence result can be obtained (see Proposition A2 in Appendix B),

(45) $$\begin{array}{c} \lim_{n\to \infty }\underset{x\in A_{{\tilde{\mu }}_{n}}}{\sup}\left|\frac{d{\tilde{\mu }}_{n}}{d\mu_{n}^{*}}\left(x\right)-1\right|=0,\;P_{\mu }\text{-}a.s.. \end{array}$$

Let A denote the collection of sequences $x_{1},x_{2},\ldots$ where the convergence in (45) is holding (this set is typical meaning that $P_{\mu }\left(A\right)=1$). The rest of the proof reduces to show that for any arbitrary ${\left(x_{n}\right)}_{n\geq 1}\in A$, its respective sequence of induced measures $\left\{\mu_{n}^{*}:n\geq 1\right\}$ (the dependency of $\mu_{n}^{*}$ on the sequence ${\left(x_{n}\right)}_{n\geq 1}$ will be considered implicit for the rest of the proof) satisfies the sufficient conditions of Lemma 3.

Let us fix an arbitrary ${\left(x_{n}\right)}_{n\geq 1}\in A$:

Weak convergence $\mu_{n}^{*}\Rightarrow \mu$: Without loss of generality we consider that $A_{{\tilde{\mu }}_{n}}=A_{v}$ for all n≥1. Since $a_{n}\to 0$ and $h_{n}\to 0$, $f_{{\tilde{\mu }}_{n}}\left(x\right)\to \mu \left(\left\{x\right\}\right)$ $\forall x\in A_{v}$, we got the weak convergence of ${\tilde{\mu }}_{n}$ to μ. On the other hand by definition of A, $\lim_{n\to \infty }\sup_{x\in A_{{\tilde{\mu }}_{n}}}\left|\frac{f_{{\tilde{\mu }}_{n}}\left(x\right)}{f_{\mu_{n}^{*}}\left(x\right)}-1\right|=0$ that implies that $\lim_{n\to \infty }\left|f_{\mu_{n}^{*}}\left(x\right)-f_{{\tilde{\mu }}_{n}}\left(x\right)\right|=0$ for all $x\in A_{v}$ and, consequently, $\mu_{n}^{*}\Rightarrow \mu$.

**The condition in (12):** By construction $\mu \ll \mu_{n}^{*}$, $\mu \ll {\tilde{\mu }}_{n}$ and ${\tilde{\mu }}_{n}\approx \mu_{n}^{*}$ for all *n*, then we will use the following equality:

(46) $$\log\frac{d\mu }{d\mu_{n}^{*}}\left(x\right)=\log\frac{d\mu }{d{\tilde{\mu }}_{n}}\left(x\right)+\log\frac{d{\tilde{\mu }}_{n}}{d\mu_{n}^{*}}\left(x\right),$$

for all $x\in A_{\mu }$. Concerning the approximation error term of (46), i.e., $\log\frac{d\mu }{d{\tilde{\mu }}_{n}}\left(x\right)$, $\forall x\in A_{\mu }$

(47) $$\frac{d{\tilde{\mu }}_{n}}{d\mu }\left(x\right)=\left(1-a_{n}\right)\left[\frac{\mu (A_{n}\left(x\right))}{\mu (\left\{x\right\})}\frac{v(\left\{x\right\})}{v(A_{n}\left(x\right))}\right]+a_{n}\frac{v(\left\{x\right\})}{\mu (\left\{x\right\})}.$$

Given that μ∈H(X|v), this is equivalent to state that $\log(\frac{d\mu }{dv}\left(x\right))$ is bounded μ-almost everywhere, which is equivalent to say that $m\equiv \inf_{x\in A_{\mu }}\frac{d\mu }{dv}\left(x\right)>0$ and $M\equiv \sup_{x\in A_{\mu }}\frac{d\mu }{dv}\left(x\right)<\infty$. From this, $\forall A\subset A_{\mu }$,

(48) mv(A)≤μ(A)≤Mv(A).

Then we have that, $\forall x\in A_{\mu }$ $\frac{m}{M}\leq \left[\frac{\mu (A_{n}\left(x\right))}{\mu (\left\{x\right\})}\frac{v(\left\{x\right\})}{v(A_{n}\left(x\right))}\right]\leq \frac{M}{m}$. Therefore for *n* sufficient large, $0<\frac{1}{2}\frac{m}{M}\leq \frac{d{\tilde{\mu }}_{n}}{d\mu }\left(x\right)\leq \frac{M}{m}+M<\infty$ for all *x* in $A_{\mu }$. Hence, there exists $N_{o}>0$ such that $\sup_{n\geq N_{o}}\sup_{x\in A_{\mu }}\left|\log\frac{d{\tilde{\mu }}_{n}}{d\mu }\left(x\right)\right|<\infty$.

For the estimation error term of (46), i.e., $\log\frac{d{\tilde{\mu }}_{n}}{d\mu_{n}^{*}}\left(x\right)$, note that from the fact that $(x_{n})\in A$, and the convergence in (45), there exists $N_{1}>0$ such that for all $n\geq N_{1}$ $\sup_{x\in A_{\mu }}\left|\log\frac{d{\tilde{\mu }}_{n}}{d\mu_{n}^{*}}\left(x\right)\right|<\infty$, given that $A_{\mu }\subset A_{{\tilde{\mu }}_{n}}=A_{v}$. Then using (46), for all $n\geq \max\left\{N_{0},N_{1}\right\}$ $\sup_{x\in A_{\mu }}\left|\log\frac{d\mu_{n}^{*}}{d\mu }\left(x\right)\right|<\infty$, which verifies (12).

**The condition in (13):** Defining the function $\phi_{n}^{*}\left(x\right)\equiv 1_{A_{v}\backslash A_{\mu }}\left(x\right)\cdot f_{\mu_{n}^{*}}\left(x\right)\log\left(1/f_{\mu_{n}^{*}}\left(x\right)\right)$, we want to verify that $\lim_{n\to \infty }\int_{X}\phi_{n}^{*}\left(x\right)d\lambda \left(x\right)=0$. Considering that $(x_{n})\in A$ for all ϵ>0, there exists N(ϵ)>0 such that $\sup_{x\in A_{{\tilde{\mu }}_{n}}}\left|\frac{f_{{\tilde{\mu }}_{n}}\left(x\right)}{f_{\mu_{n}^{*}}\left(x\right)}-1\right|<\epsilon$ and then

(49) $$\left(1-\epsilon \right)f_{{\tilde{\mu }}_{n}}\left(x\right)<f_{\mu_{n}^{*}}\left(x\right)<\left(1+\epsilon \right)f_{{\tilde{\mu }}_{n}}\left(x\right),\;\mathrm{for}\;\mathrm{all}\;x\in A_{v}.$$

From (49), $0\leq \phi_{n}^{*}\left(x\right)\leq \left(1+\epsilon \right)f_{{\tilde{\mu }}_{n}}\left(x\right)\log\left(1/\left(1-\epsilon \right)f_{{\tilde{\mu }}_{n}}\left(x\right)\right)$ for all n≥N(ϵ). Analyzing $f_{{\tilde{\mu }}_{n}}\left(x\right)$ in (44), there are two scenarios: $A_{n}\left(x\right)\cap A_{\mu }=\emptyset$ where $f_{{\tilde{\mu }}_{n}}\left(x\right)=a_{n}f_{v}\left(x\right)$ and, otherwise, $f_{{\tilde{\mu }}_{n}}\left(x\right)=f_{v}\left(x\right)\left(a_{n}+\left(1-a_{n}\right)\mu \left(A_{n}\left(x\right)\cap A_{\mu }\right)/v\left(A_{n}\left(x\right)\right)\right)$. Let us define:

(50) $$\begin{array}{c} B_{n}\equiv \left\{x\in A_{v}\backslash A_{\mu }:A_{n}\left(x\right)\cap A_{\mu }=\emptyset \right\}\;\mathrm{and}\;C_{n}\equiv \left\{x\in A_{v}\backslash A_{\mu }:A_{n}\left(x\right)\cap A_{\mu }\neq \emptyset \right\}. \end{array}$$

Then for all n≥N(ϵ),

(51) $$\begin{array}{cc} \sum_{x\in X}\phi_{n}^{*}\left(x\right) & \leq \sum_{x\in A_{v}\backslash A_{\mu }}\left(1+\epsilon \right)f_{{\tilde{\mu }}_{n}}\left(x\right)\log1/\left(\left(1-\epsilon \right)f_{{\tilde{\mu }}_{n}}\left(x\right)\right)\\ & =\sum_{x\in B_{n}}\left(1+\epsilon \right)a_{n}f_{v}\left(x\right)\log\frac{1}{\left(1-\epsilon \right)a_{n}f_{v}\left(x\right)}+\sum_{x\in X}{\tilde{\phi }}_{n}\left(x\right), \end{array}$$

with ${\tilde{\phi }}_{n}\left(x\right)\equiv 1_{C_{n}}\left(x\right)\cdot \left(1+\epsilon \right)f_{{\tilde{\mu }}_{n}}\left(x\right)\log\frac{1}{\left(1-\epsilon \right)f_{{\tilde{\mu }}_{n}}\left(x\right)}$. The left term in (51) is upper bounded by $a_{n}\left(1+\epsilon \right)\left(H\left(v\right)+\log\left(1/a_{n}\right)\right)$, which goes to zero with *n* from $\left(a_{n}\right)$ being o(1) and the fact that v∈H(X). For the right term in (51), $\left(h_{n}\right)$ being o(1) implies that *x* belongs to $B_{n}$ eventually (in *n*) $\forall x\in A_{v}\backslash A_{\mu }$, then ${\tilde{\phi }}_{n}\left(x\right)$ tends to zero point-wise as *n* goes to infinity. On the other hand, for all $x\in C_{n}$ (see (50)), we have that

(52) $$\begin{array}{c} \frac{1}{1/m+1}\leq \frac{\mu (A_{n}\left(x\right)\cap A_{\mu })}{v\left(A_{n}\left(x\right)\cap A_{\mu }\right)+v\left(A_{v}\backslash A_{\mu }\right)}\leq \frac{\mu (A_{n}\left(x\right))}{v(A_{n}\left(x\right))}\leq \frac{\mu (A_{n}\left(x\right)\cap A_{\mu })}{v(A_{n}\left(x\right)\cap A_{\mu })}\leq M. \end{array}$$

These inequalities derive from (48). Consequently for all x∈X, if *n* sufficiently large such that $a_{n}<0.5$, then

(53) $$\begin{array}{cc} 0\leq {\tilde{\phi }}_{n}\left(x\right) & \leq \left(1+\epsilon \right)\left(a_{n}+\left(1-a_{n}\right)M\right)f_{v}\left(x\right)\log\frac{1}{\left(1-\epsilon \right)(a_{n}+\left(1-a_{n}\right)m/\left(m+1\right))}\\ & \leq \left(1+\epsilon \right)\left(1+M\right)f_{v}\left(x\right)\left[\log\frac{2(m+1)}{\left(1-\epsilon \right)}+\log\frac{1}{f_{v}\left(x\right)}\right]. \end{array}$$

Hence from (50), ${\tilde{\phi }}_{n}\left(x\right)$ is bounded by a fix function that is $\ell_{1}\left(X\right)$ by the assumption that v∈H(X). Then by the dominated convergence theorems [43] and (51),

$$\lim_{n\to \infty }\sum_{X}\phi_{n}^{*}\left(x\right)\leq \lim_{n\to \infty }\sum_{X}{\tilde{\phi }}_{n}\left(x\right).$$

In summary, we have shown that for any arbitrary $(x_{n})\in A$ the sufficient conditions of Lemma 3 are satisfied, which proves the result in (25) reminding that $P_{\mu }\left(A\right)=1$ from (45). ☐

**Proof of Theorem** **4.** Let us first introduce the oracle probability

(54) $$\mu_{\epsilon_{n}}\equiv \mu \left(\cdot |\Gamma_{\epsilon_{n}}\right)\in P\left(X\right).$$

Note that $\mu_{\epsilon_{n}}$ is a random probability measure (function of the i.i.d sequence $X_{1},\ldots ,X_{n}$) as $\Gamma_{\epsilon_{n}}$ is a data-driven set, see (26). We will first show that:

(55) $$\lim_{n\to \infty }H\left(\mu_{\epsilon_{n}}\right)=H\left(\mu \right)\;\mathrm{and}\;\lim_{n\to \infty }D(\mu_{\epsilon_{n}}\left||\mu \right)=0,\;P_{\mu }\text{-}a.s.$$

Under the assumption on $\left(\epsilon_{n}\right)$ of Theorem 4, $\lim_{n\to \infty }\left|\mu \left(\Gamma_{\epsilon_{n}}\right)-{\hat{\mu }}_{n}\left(\Gamma_{\epsilon_{n}}\right)\right|=0$, $P_{\mu }$-a.s. (this result derives from the fact that $\lim_{n\to \infty }V\left(\mu /\sigma_{\epsilon_{n}},{\hat{\mu }}_{n}/\sigma_{\epsilon_{n}}\right)=0$, $P_{\mu }$-a.s. , from (63)) In addition, since $\left(\epsilon_{n}\right)$ is o(1) then $\lim_{n\to \infty }{\hat{\mu }}_{n}\left(\Gamma_{\epsilon_{n}}\right)=1$, which implies that $\lim_{n\to \infty }\mu \left(\Gamma_{\epsilon_{n}}\right)=1$ $P_{\mu }$-a.s. From this $\mu_{\epsilon_{n}}\Rightarrow \mu$, $P_{\mu }$-a.s. Let us consider a sequences $\left(x_{n}\right)$ where $\lim_{n\to \infty }\mu \left(\Gamma_{\epsilon_{n}}\right)=1$. Constrained to that

(56) $$\lim\underset{n\to \infty }{\sup}\underset{x\in A_{\mu }}{\sup}\frac{f_{\mu_{\epsilon_{n}}}\left(x\right)}{f_{\mu }\left(x\right)}=\lim\underset{n\to \infty }{\sup}\frac{1}{\mu (\Gamma_{\epsilon_{n}})}<\infty .$$

Then there is N>0 such that $\sup_{n>N}\sup_{x\in A_{\mu }}\frac{f_{\mu_{\epsilon_{n}}}\left(x\right)}{f_{\mu }\left(x\right)}<\infty$. Hence from Lemma 2, $\lim_{n\to \infty }D(\mu_{\epsilon_{n}}\left||\mu \right)=$ 0 and $\lim_{n\to \infty }\left|H\left(\mu_{\epsilon_{n}}\right)-H\left(\mu \right)\right|=0$. Finally, the set of sequences $\left(x_{n}\right)$ where $\lim_{n\to \infty }\mu \left(\Gamma_{\epsilon_{n}}\right)=1$ has probability one (with respect to $P_{\mu }$), which proves (55).

For the rest of the proof, we concentrate on the analysis of $\left|H\left({\hat{\mu }}_{n,\epsilon_{n}}\right)-H\left(\mu_{\epsilon_{n}}\right)\right|$ that can be attributed to the estimation error aspect of the problem. It is worth noting that by construction $A_{{\hat{\mu }}_{n,\epsilon_{n}}}=A_{\mu_{\epsilon_{n}}}=\Gamma_{\epsilon_{n}}$, $P_{\mu }$-a.s.. Consequently, we can use

(57) $$H\left({\hat{\mu }}_{n,\epsilon_{n}}\right)-H\left(\mu_{\epsilon_{n}}\right)=\sum_{x\in \Gamma_{\epsilon_{n}}}\left[\mu_{\epsilon_{n}}\left(\left\{x\right\}\right)-{\hat{\mu }}_{n,\epsilon_{n}}\left(\left\{x\right\}\right)\right]\log{\hat{\mu }}_{n,\epsilon_{n}}\left(\left\{x\right\}\right)+D(\mu_{\epsilon_{n}}\left|\right|{\hat{\mu }}_{n,\epsilon_{n}}).$$

The first term on the RHS of (57) is upper bounded by $\log1/m_{{\hat{\mu }}_{n}}^{\epsilon_{n}}\cdot V\left(\mu_{\epsilon_{n}},{\hat{\mu }}_{n,\epsilon_{n}}\right)$ $\leq \log1/\epsilon_{n}\cdot V\left(\mu_{\epsilon_{n}},{\hat{\mu }}_{n,\epsilon_{n}}\right)$. Concerning the second term on the RHS of (57), it is possible to show (details presented in Appendix C) that

(58) $$D(\mu_{\epsilon_{n}}\left|\right|{\hat{\mu }}_{n,\epsilon_{n}})\leq \frac{2\log\frac{e}{\epsilon_{n}}}{\mu (\Gamma_{\epsilon_{n}})}\cdot V\left(\mu /\sigma_{\epsilon_{n}},{\hat{\mu }}_{n}/\sigma_{\epsilon_{n}}\right),$$

where

(59) $$V\left(\mu /\sigma_{\epsilon_{n}},{\hat{\mu }}_{n}/\sigma_{\epsilon_{n}}\right)\equiv \underset{A\in \sigma_{\epsilon_{n}}}{\sup}\left|\mu \left(A\right)-{\hat{\mu }}_{n}\left(A\right)\right|.$$

In addition, it can be verified (details presented in Appendix D) that

(60) $$V\left(\mu_{\epsilon_{n}},{\hat{\mu }}_{n,\epsilon_{n}}\right)\leq K\cdot V\left(\mu /\sigma_{\epsilon_{n}},{\hat{\mu }}_{n}/\sigma_{\epsilon_{n}}\right),$$

for some universal constant K>0. Therefore from (57), (58) and (60), there is C>0 such that

(61) $$\left|H\left({\hat{\mu }}_{n,\epsilon_{n}}\right)-H\left(\mu_{\epsilon_{n}}\right)\right|\leq \frac{C}{\mu (\Gamma_{\epsilon_{n}})}\log\frac{1}{\epsilon_{n}}\cdot V\left(\mu /\sigma_{\epsilon_{n}},{\hat{\mu }}_{n}/\sigma_{\epsilon_{n}}\right).$$

As mentioned before, $\mu (\Gamma_{\epsilon_{n}})$ goes to 1 almost surely, then we need to concentrate on the analysis of the asymptotic behavior of $\log1/\epsilon_{n}\cdot V\left(\mu /\sigma_{\epsilon_{n}},{\hat{\mu }}_{n}/\sigma_{\epsilon_{n}}\right)$. From Hoeffding’s inequality [28], we have that ∀δ>0

(62) $$P_{\mu }^{n}\left(\log1/\epsilon_{n}\cdot V\left(\mu /\sigma_{\epsilon_{n}},{\hat{\mu }}_{n}/\sigma_{\epsilon_{n}}\right)>\delta \right)\leq 2^{\left|\Gamma_{\epsilon_{n}}\right|+1}\cdot e^{-\frac{2n\delta^{2}}{{\left(\log1/\epsilon_{n}\right)}^{2}}},$$

considering that by construction $\left|\sigma_{\epsilon_{n}}\right|\leq 2^{\left|\Gamma_{\epsilon_{n}}\right|+1}\leq 2^{1/\epsilon_{n}+1}$. Assuming that $\left(\epsilon_{n}\right)$ is $O(n^{-\tau })$,

$$\ln P_{\mu }^{n}\left(\log1/\epsilon_{n}\cdot V\left(\mu /\sigma_{\epsilon_{n}},{\hat{\mu }}_{n}/\sigma_{\epsilon_{n}}\right)>\delta \right)\leq \left(n^{\tau }+1\right)\ln2-\frac{2n\delta^{2}}{\tau \log n}.$$

Therefore for all τ∈(0,1), δ>0 and any arbitrary l∈(τ,1)

(63) $$\lim\underset{n\to \infty }{\sup}\frac{1}{n^{l}}\cdot \ln P_{\mu }^{n}\left(\log1/\epsilon_{n}\cdot V\left(\mu /\sigma_{\epsilon_{n}},{\hat{\mu }}_{n}/\sigma_{\epsilon_{n}}\right)>\delta \right)<0.$$

This last result is sufficient to show that $\sum_{n\geq 1}P_{\mu }^{n}\left(\log1/\epsilon_{n}\cdot V\left(\mu /\sigma_{\epsilon_{n}},{\hat{\mu }}_{n}/\sigma_{\epsilon_{n}}\right)>\delta \right)<\infty$ that concludes the argument from the Borel-Cantelli Lemma. ☐

**Proof of Theorem** **5.** We consider the expression

(64) $$\left|H\left(\mu \right)-H\left({\hat{\mu }}_{n,\epsilon_{n}}\right)\right|\leq \left|H\left(\mu \right)-H\left(\mu_{\epsilon_{n}}\right)\right|+\left|H\left(\mu_{\epsilon_{n}}\right)-H\left({\hat{\mu }}_{n,\epsilon_{n}}\right)\right|$$

to analize the approximation error and the estimation error terms separately.

• Approximation Error Analysis

Note that $\left|H\left(\mu \right)-H\left(\mu_{\epsilon_{n}}\right)\right|$ is a random object as $\mu_{\epsilon_{n}}$ in (54) is a function of the data-dependent partition and, consequently, a function of $X_{1},\ldots ,X_{n}$. In the following, we consider the oracle set

(65) $${\tilde{\Gamma }}_{\epsilon_{n}}\equiv \left\{x\in X:\mu \left(\left\{x\right\}\right)\geq \epsilon_{n}\right\},$$

and the oracle conditional probability

(66) $${\tilde{\mu }}_{\epsilon_{n}}\equiv \mu \left(\cdot |{\tilde{\Gamma }}_{\epsilon_{n}}\right)\in P.\left(X\right).$$

Note that ${\tilde{\Gamma }}_{\epsilon_{n}}$ is a deterministic function of $\left(\epsilon_{n}\right)$ and so is the measure ${\tilde{\mu }}_{\epsilon_{n}}$ in (66). From definitions and triangular inequality:

(67) $$\begin{array}{cc} \left|H\left(\mu \right)-H\left({\tilde{\mu }}_{\epsilon_{n}}\right)\right| & \leq \sum_{x\in {\tilde{\Gamma }}_{\epsilon_{n}}^{c}}\mu \left(\left\{x\right\}\right)\log\frac{1}{\mu (\left\{x\right\})}+\log\frac{1}{\mu ({\tilde{\Gamma }}_{\epsilon_{n}})}\\ & +\left(\frac{1}{\mu ({\tilde{\Gamma }}_{\epsilon_{n}})}-1\right)\cdot \sum_{x\in {\tilde{\Gamma }}_{\epsilon_{n}}}\mu \left(\left\{x\right\}\right)\log\frac{1}{\mu (\left\{x\right\})}, \end{array}$$

and, similarly, the approximation error is bounded by

(68) $$\begin{array}{cc} \left|H\left(\mu \right)-H\left(\mu_{\epsilon_{n}}\right)\right| & \leq \sum_{x\in \Gamma_{\epsilon_{n}}^{c}}\mu \left(\left\{x\right\}\right)\log\frac{1}{\mu (\left\{x\right\})}\\ & +\log\frac{1}{\mu (\Gamma_{\epsilon_{n}})}+\left(\frac{1}{\mu (\Gamma_{\epsilon_{n}})}-1\right)\cdot \sum_{x\in \Gamma_{\epsilon_{n}}}\mu \left(\left\{x\right\}\right)\log\frac{1}{\mu (\left\{x\right\})}. \end{array}$$

We denote the RHS of (67) and (68) by $a_{\epsilon_{n}}$ and $b_{\epsilon_{n}}\left(X_{1},\ldots ,X_{n}\right)$, respectively.

We can show that if $\left(\epsilon_{n}\right)$ is $O(n^{-\tau })$ and τ∈(0,1/2), then

(69) $$\lim\underset{n\to \infty }{\sup}b_{\epsilon_{n}}\left(X_{1},\ldots ,X_{n}\right)-a_{2\epsilon_{n}}\leq 0,\;P_{\mu }\text{-}a.s.,$$

which from (68) implies that $\left|H\left(\mu \right)-H\left(\mu_{\epsilon_{n}}\right)\right|$ is $O(a_{2\epsilon_{n}})$, $P_{\mu }$-a.s. The proof of (69) is presented in Appendix E.

Then, we need to analyze the rate of convergence of the deterministic sequence $\left(a_{2\epsilon_{n}}\right)$. Analyzing the RHS of (67), we recognize two independent terms: the partial entropy sum $\sum_{x\in {\tilde{\Gamma }}_{\epsilon_{n}}^{c}}\mu \left(\left\{x\right\}\right)\log\frac{1}{\mu (\left\{x\right\})}$ and the rest that is bounded asymptotically by $\mu \left({\tilde{\Gamma }}_{\epsilon_{n}}^{c}\right)\left(1+H\left(\mu \right)\right)$, using the fact that lnx≤x−1 for x≥1. Here is where the tail condition on μ plays a role. From the tail condition, we have that

(70) $$\begin{array}{cc} \mu ({\tilde{\Gamma }}_{\epsilon_{n}}^{c}) & \leq \mu \left(\left\{{\left(k_{o}/\epsilon_{n}\right)}^{1/p}+1,{\left(k_{o}/\epsilon_{n}\right)}^{1/p}+2,{\left(k_{o}/\epsilon_{n}\right)}^{1/p}+3,\ldots \right\}\right)=\sum_{x\geq {\left(\frac{k_{o}}{\epsilon_{n}}\right)}^{1/p}+1}\mu \left(\left\{x\right\}\right)\\ & \leq k_{1}\cdot S_{{\left(\frac{k_{o}}{\epsilon_{n}}\right)}^{1/p}+1}, \end{array}$$

where $S_{x_{o}}\equiv \sum_{x\geq x_{o}}x^{-p}$. Similarly as $\left\{0,1,\ldots ,{\left(k_{o}/\epsilon_{n}\right)}^{1/p}\right\}\subset {\tilde{\Gamma }}_{\epsilon_{n}}$, then

(71) $$\begin{array}{cc} \sum_{x\in {\tilde{\Gamma }}_{\epsilon_{n}}^{c}}\mu \left(\left\{x\right\}\right)\log\frac{1}{\mu (\left\{x\right\})} & \leq \sum_{x\geq {\left(\frac{k_{o}}{\epsilon_{n}}\right)}^{1/p}+1}\mu \left(\left\{x\right\}\right)\log\frac{1}{\mu (\left\{x\right\})}\leq \sum_{x\geq {\left(\frac{k_{o}}{\epsilon_{n}}\right)}^{1/p}+1}k_{1}x^{-p}\cdot \log\frac{1}{k_{0}x^{-p}}\\ & \leq k_{1}\log p\cdot R_{{\left(\frac{k_{o}}{\epsilon_{n}}\right)}^{1/p}+1}+k_{1}\log1/k_{0}\cdot S_{{\left(\frac{k_{o}}{\epsilon_{n}}\right)}^{1/p}+1}, \end{array}$$

where $R_{x_{o}}\equiv \sum_{x\geq x_{o}}x^{-p}\log x$.

In Appendix F, it is shown that $S_{x_{o}}\leq C_{0}\cdot x_{o}^{1-p}$ and $R_{x_{o}}\leq C_{1}\cdot x_{o}^{1-p}$ for constants $C_{1}>0$ and $C_{0}>0$. Integrating these results in the RHS of (70) and (71) and considering that $\left(\epsilon_{n}\right)$ is $O(n^{-\tau })$, we have that both $\mu ({\tilde{\Gamma }}_{\epsilon_{n}}^{c})$ and $\sum_{x\in {\tilde{\Gamma }}_{\epsilon_{n}}^{c}}\mu \left(\left\{x\right\}\right)\log\frac{1}{\mu (\left\{x\right\})}$ are $O(n^{-\frac{\tau (p-1)}{p}})$. This implies that our oracle sequence $\left(a_{\epsilon_{n}}\right)$ is $O(n^{-\frac{\tau (p-1)}{p}})$.

In conclusion, if $\epsilon_{n}$ is $O(n^{-\tau })$ for τ∈(0,1/2), it follows that

(72) $$\left|H\left(\mu \right)-H\left(\mu_{\epsilon_{n}}\right)\right|\;\mathrm{is}\;O\left(n^{-\frac{\tau (p-1)}{p}}\right),\;P_{\mu }\text{-}a.s.$$

• Estimation Error Analysis

Let us consider $\left|H\left(\mu_{\epsilon_{n}}\right)-H\left({\hat{\mu }}_{n,\epsilon_{n}}\right)\right|$. From the bound in (61) and the fact that for any τ∈(0,1), $\lim_{n\to \infty }\mu (\Gamma_{\epsilon_{n}})=1$ $P_{\mu }$-a.s. from (63), the problem reduces to analyze the rate of convergence of the following random object:

(73) $$\rho_{n}\left(X_{1},\ldots ,X_{n}\right)\equiv \log\frac{1}{\epsilon_{n}}\cdot V\left(\mu /\sigma \left(\Gamma_{\epsilon_{n}}\right),{\hat{\mu }}_{n}/\sigma \left(\Gamma_{\epsilon_{n}}\right)\right).$$

We will analize, instead, the oracle version of $\rho_{n}\left(X_{1},\ldots ,X_{n}\right)$ given by:

(74) $$\xi_{n}\left(X_{1},\ldots ,X_{n}\right)\equiv \log\frac{1}{\epsilon_{n}}\cdot V\left(\mu /\sigma \left({\tilde{\Gamma }}_{\epsilon_{n}/2}\right),{\hat{\mu }}_{n}/\sigma \left({\tilde{\Gamma }}_{\epsilon_{n}/2}\right)\right),$$

where ${\tilde{\Gamma }}_{\epsilon }\equiv \left\{x\in X:\mu (\left\{x\right\})\geq \epsilon \right\}$ is the oracle counterpart of $\Gamma_{\epsilon }$ in (26). To do so, we can show that if $\epsilon_{n}$ is $O(n^{-\tau })$ with τ∈(0,1/2), then

(75) $$\lim\underset{n\to \infty }{\inf}\xi_{n}\left(X_{1},\ldots ,X_{n}\right)-\rho_{n}\left(X_{1},\ldots ,X_{n}\right)\geq 0,\;\;P_{\mu }\text{-}a.s..$$

The proof of (75) is presented in Appendix G.

Moving to the almost sure rate of convergence of $\xi_{n}\left(X_{1},\ldots ,X_{n}\right)$, it is simple to show for our *p*-power dominating distribution that if $\left(\epsilon_{n}\right)$ is $O(n^{-\tau })$ and τ∈(0,p) then

$$\lim_{n\to \infty }\xi_{n}\left(X_{1},\ldots ,X_{n}\right)=0\;P_{\mu }\text{-}a.s.,$$

and, more specifically,

(76) $$\xi_{n}\left(X_{1},\ldots ,X_{n}\right)\;\mathrm{is}\;o\left(n^{-q}\right)\;\mathrm{for}\;\mathrm{all}\;q\in \left(0,\left(1-\tau /p\right)/2\right),\;P_{\mu }\text{-}a.s..$$

The argument is presented in Appendix H.

In conclusion, if $\epsilon_{n}$ is $O(n^{-\tau })$ for τ∈(0,1/2), it follows that

(77) $$\left|H\left(\mu_{\epsilon_{n}}\right)-H\left({\hat{\mu }}_{n,\epsilon_{n}}\right)\right|\;\mathrm{is}\;O\left(n^{-q}\right),\;P_{\mu }\text{-}a.s.,$$

for all q∈(0,(1−τ/p)/2).

• Estimation vs. Approximation Errors

Coming back to (64) and using (72) and (77), the analysis reduces to finding the solution $\tau^{*}$ in (0,1/2) that offers the best trade-off between the estimation and approximation error rate:

(78) $$\tau^{*}\equiv \arg\max_{\tau (0,1/2)}\min\left\{\frac{\left(1-\tau /p\right)}{2},\frac{\tau (p-1)}{p}\right\}.$$

It is simple to verify that $\tau^{*}=1/2$. Then by considering τ arbitrary close to the admissible limit 1/2, we can achieve a rate of convergence for $\left|H\left(\mu \right)-H\left({\hat{\mu }}_{n,\epsilon_{n}}\right)\right|$ that is arbitrary close to $O(n^{-\frac{1}{2}\left(1-1/p\right)})$, P-a.s.

More formally, for any $l\in (0,\frac{1}{2}\left(1-1/p\right))$ we can take $\tau \in (\frac{l}{\left(1-1/p\right)},\frac{1}{2})$ where $\left|H\left(\mu \right)-H\left({\hat{\mu }}_{n,\epsilon_{n}}\right)\right|$ is $o(n^{-l})$, $P_{\mu }$-a.s., from (72) and (77).

Finally, a simple corollary of this analysis is to consider $\tau \left(p\right)=\frac{1}{2+1/p}<1/2$ where:

(79) $$\left|H\left(\mu \right)-H\left({\hat{\mu }}_{n,\epsilon_{n}}\right)\right|\;\mathrm{is}\;O\left(n^{-\frac{1-1/p}{2+1/p}}\right),\;P_{\mu }\text{-}a.s.,$$

which concludes the argument. ☐

**Proof of Theorem** **6.** The argument follows the proof of Theorem 5. In particular, we use the estimation-approximation error bound:

(80) $$\left|H\left(\mu \right)-H\left({\hat{\mu }}_{n,\epsilon_{n}}\right)\right|\leq \left|H\left(\mu \right)-H\left(\mu_{\epsilon_{n}}\right)\right|+\left|H\left(\mu_{\epsilon_{n}}\right)-H\left({\hat{\mu }}_{n,\epsilon_{n}}\right)\right|,$$

and the following two results derived in the proof of Theorem 5: If $\left(\epsilon_{n}\right)$ is $O(n^{-\tau })$ with τ∈(0,1/2) then (for the approximation error)

(81) $$\left|H\left(\mu \right)-H\left(\mu_{\epsilon_{n}}\right)\right|\;\mathrm{is}\;O\left(a_{2\epsilon_{n}}\right)\;P_{\mu }\text{-}a.s.,$$

with $a_{\epsilon_{n}}=\sum_{x\in {\tilde{\Gamma }}_{\epsilon_{n}}^{c}}\mu \left(\left\{x\right\}\right)\log\frac{1}{\mu (\left\{x\right\})}+\mu \left({\tilde{\Gamma }}_{\epsilon_{n}}^{c}\right)\left(1+H\left(\mu \right)\right),$ while (for the estimation error)

(82) $$\left|H\left(\mu_{\epsilon_{n}}\right)-H\left({\hat{\mu }}_{n,\epsilon_{n}}\right)\right|\;\mathrm{is}\;O\left(\xi_{n}\left(X_{1},\ldots ,X_{n}\right)\right)\;P_{\mu }\text{-}a.s.,$$

with $\xi_{n}\left(X_{1},\ldots ,X_{n}\right)=\log\frac{1}{\epsilon_{n}}\cdot V\left(\mu /\sigma \left({\tilde{\Gamma }}_{\epsilon_{n}/2}\right),{\hat{\mu }}_{n}/\sigma \left({\tilde{\Gamma }}_{\epsilon_{n}/2}\right)\right).$

For the estimation error, we need to bound the rate of convergence of $\xi_{n}\left(X_{1},\ldots ,X_{n}\right)$ to zero almost surely. We first note that $\left\{1,\ldots ,x_{o}\left(\epsilon_{n}\right)\right\}={\tilde{\Gamma }}_{\epsilon_{n}}$ with $x_{o}\left(\epsilon_{n}\right)=\left\lfloor 1/\alpha \ln\left(k_{0}/\epsilon_{n}\right)\right\rfloor$. Then from Hoeffding’s inequality we have that

(83) $$\begin{array}{cc} P_{\mu }^{n}\left(\left\{\xi_{n}\left(X_{1},\ldots ,X_{n}\right)>\delta \right\}\right) & \leq 2^{\left|({\tilde{\Gamma }}_{\epsilon_{n}/2})\right|}\cdot e^{-2n\frac{\delta^{2}}{\log{\left(1/\epsilon_{n}\right)}^{2}}}\\ & \leq 2^{1/\alpha \ln(2k_{0}/\epsilon_{n})+1}\cdot e^{-2n\frac{\delta^{2}}{\log{\left(1/\epsilon_{n}\right)}^{2}}}. \end{array}$$

Considering $\epsilon_{n}=O\left(n^{-\tau }\right)$, an arbitrary sequence $\left(\delta_{n}\right)$ being o(1) and l>0, it follows from (83) that

(84) $$\begin{array}{cc} \frac{1}{n^{l}}\cdot \ln P_{\mu }^{n}\left(\left\{\xi_{n}\left(X_{1},\ldots ,X_{n}\right)>\delta_{n}\right\}\right) & \leq \frac{1}{n^{l}}\ln\left(2\right)\left[1/\alpha \ln(2k_{0}/\epsilon_{n})+1\right]-n^{1-l}\frac{\delta_{n}^{2}}{\log{\left(1/\epsilon_{n}\right)}^{2}}. \end{array}$$

We note that the first term in the RHS of (84) is $O(\frac{1}{n^{l}}\log n)$ and goes to zero for all l>0, while the second term is $O(n^{1-l}\frac{\delta_{n}^{2}}{\log n^{2}})$. If we consider $\delta_{n}=O\left(n^{-q}\right)$, this second term is $O(n^{1-2q-l}\cdot \frac{1}{\log n^{2}})$. Therefore, for any q∈(0,1/2) we can take an arbitrary l∈(0,1−2q] such that $P_{\mu }^{n}\left(\left\{\xi_{n}\left(X_{1},\ldots ,X_{n}\right)>\delta_{n}\right\}\right)$ is $O(e^{-n^{l}})$ from (84). This result implies, from the Borel-Cantelli Lemma, that $\xi_{n}\left(X_{1},\ldots ,X_{n}\right)$ is $o(\delta_{n})$, $P_{\mu }$-a.s, which in summary shows that $\left|H\left(\mu_{\epsilon_{n}}\right)-H\left({\hat{\mu }}_{n,\epsilon_{n}}\right)\right|$ is $O(n^{-q})$ for all q∈(0,1/2).

For the approximation error, it is simple to verify that:

(85) $$\mu \left({\tilde{\Gamma }}_{\epsilon_{n}}^{c}\right)\leq k_{1}\cdot \sum_{x\geq x_{o}\left(\epsilon_{n}\right)+1}e^{-\alpha x}=k_{1}\cdot {\tilde{S}}_{x_{o}\left(\epsilon_{n}\right)+1}$$

and

(86) $$\begin{array}{cc} \sum_{x\in {\tilde{\Gamma }}_{\epsilon_{n}}^{c}}\mu \left(\left\{x\right\}\right)\log\frac{1}{\mu (\left\{x\right\})} & \leq \sum_{x\geq x_{o}\left(\epsilon_{n}\right)+1}k_{1}e^{-\alpha x}\log\frac{1}{k_{0}e^{-\alpha x}}=k_{1}\log\frac{1}{k_{0}}\cdot {\tilde{S}}_{x_{o}\left(\epsilon_{n}\right)+1}\\ & +\alpha \log e\cdot k_{1}\cdot {\tilde{R}}_{x_{o}\left(\epsilon_{n}\right)+1}, \end{array}$$

where ${\tilde{S}}_{x_{o}}\equiv \sum_{x\geq x_{o}}e^{-\alpha x}$ and ${\tilde{R}}_{x_{o}}\equiv \sum_{x\geq x_{o}}x\cdot e^{-\alpha x}$. At this point, it is not difficult to show that ${\tilde{S}}_{x_{o}}\leq M_{1}e^{-\alpha x_{o}}$ and ${\tilde{R}}_{x_{o}}\leq M_{2}e^{-\alpha x_{o}}\cdot x_{o}$ for some constants $M_{1}>0$ and $M_{2}>0$. Integrating these partial steps, we have that

(87) $$\begin{array}{c} a_{\epsilon_{n}}\leq k_{1}\left(1+H\left(\mu \right)+\log\frac{1}{k_{0}}\right)\cdot {\tilde{S}}_{x_{o}\left(\epsilon_{n}\right)+1}+\alpha \log e\cdot k_{1}\cdot {\tilde{R}}_{x_{o}\left(\epsilon_{n}\right)+1}\leq O_{1}\cdot \epsilon_{n}+O_{2}\cdot \epsilon_{n}\log\frac{1}{\epsilon_{n}} \end{array}$$

for some constant $O_{1}>0$ and $O_{2}>0$. The last step is from the evaluation of $x_{o}\left(\epsilon_{n}\right)=\left\lfloor 1/\alpha \ln\left(k_{0}/\epsilon_{n}\right)\right\rfloor$. Therefore from (81) and (87), it follows that $\left|H\left(\mu \right)-H\left(\mu_{\epsilon_{n}}\right)\right|$ is $O(n^{-\tau }\log n)$ $P_{\mu }$-a.s. for all τ∈(0,1/2).

The argument concludes by integrating in (80) the almost sure convergence results obtained for the estimation and approximation errors. ☐

**Proof of Theorem** **7.** Let us define the event

(88) $$B_{n}^{\epsilon }=\left\{x^{n}\in X^{n}:\Gamma_{\epsilon }\left(x^{n}\right)=A_{\mu }\right\},$$

that represents the detection of the support of μ from the data for a given ϵ>0 in (26). Note that the dependency on the data for $\Gamma_{\epsilon }$ is made explicit in this notation. In addition, let us consider the deviation event

(89) $$A_{n}^{\epsilon }\left(\mu \right)=\left\{x^{n}\in X^{n}:V\left(\mu ,{\hat{\mu }}_{n}\right)>\epsilon \right\}.$$

By the hypothesis that $\left|A_{\mu }\right|<\infty$, then $m_{\mu }=\min_{x\in A_{\mu }}f_{\mu }\left(x\right)>0$. Therefore if $x^{n}\in {\left(A_{n}^{m_{\mu }/2}\left(\mu \right)\right)}^{c}$ then ${\hat{\mu }}_{n}\left(\left\{x\right\}\right)\geq m_{\mu }/2$ for all $x\in A_{\mu }$, which implies that ${\left(B_{n}^{\epsilon }\right)}^{c}\subset A_{n}^{m_{\mu }/2}\left(\mu \right)$ as long as $0<\epsilon \leq m_{\mu }/2$. Using the hypothesis that $\epsilon_{n}\to 0$, there is N>0 such that for all n≥N ${\left(B_{n}^{\epsilon_{n}}\right)}^{c}\subset A_{n}^{m_{\mu }/2}\left(\mu \right)$ and, consequently,

(90) $$P_{\mu }^{n}\left({\left(B_{n}^{\epsilon_{n}}\right)}^{c}\right)\leq P_{\mu }^{n}\left(A_{n}^{m_{\mu }/2}\left(\mu \right)\right)\leq 2^{k+1}\cdot e^{-\frac{nm_{\mu }^{2}}{4}},$$

the last from Hoeffding’s inequality considering $k=\left|A_{\mu }\right|<\infty$.

If we consider the events:

(91) $$\begin{array}{cc} C_{n}^{\epsilon }\left(\mu \right) & =\left\{x^{n}\in X^{n}:\left|H\left({\hat{\mu }}_{n,\epsilon_{n}}\right)-H\left(\mu \right)\right|>\epsilon \right\}\;\mathrm{and}\; \end{array}$$

(92) $$\begin{array}{cc} D_{n}^{\epsilon }\left(\mu \right) & =\left\{x^{n}\in X^{n}:\left|H\left({\hat{\mu }}_{n}\right)-H\left(\mu \right)\right|>\epsilon \right\} \end{array}$$

and we use the fact that by definition ${\hat{\mu }}_{n,\epsilon_{n}}={\hat{\mu }}_{n}$ conditioning on $B_{n}^{\epsilon_{n}}$, it follows that $C_{n}^{\epsilon }\left(\mu \right)\cap B_{n}^{\epsilon_{n}}\subset D_{n}^{\epsilon }\left(\mu \right)$. Then, for all ϵ>0 and n≥N

(93) $$\begin{array}{cc} P_{\mu }^{n}\left(C_{n}^{\epsilon }\left(\mu \right)\right) & \leq P_{\mu }^{n}\left(C_{n}^{\epsilon }\left(\mu \right)\cap B_{n}^{\epsilon_{n}}\right)+P_{\mu }^{n}\left({\left(B_{n}^{\epsilon_{n}}\right)}^{c}\right)\\ & \leq P_{\mu }^{n}\left(D_{n}^{\epsilon }\left(\mu \right)\right)+P_{\mu }^{n}\left({\left(B_{n}^{\epsilon_{n}}\right)}^{c}\right)\\ & \leq 2^{k+1}\left[e^{-\frac{2n\epsilon^{2}}{{\left(M_{\mu }+\frac{\log e}{m_{\mu }}\right)}^{2}}}+e^{-\frac{nm_{\mu }^{2}}{4}}\right], \end{array}$$

the last inequality from Theorem 1 and (90). ☐

## Appendix A. Minimax risk for Finite Entropy Distributions in ∞-Alphabets

**Proposition** **A1.** $R_{n}^{*}=\infty .$

For the proof, we use the following lemma that follows from [26] (Theorem 1).

**Lemma** **A1.** Let us fix two arbitrary real numbers δ>0 and ϵ>0. Then there are P, Q two finite supported distributions on H(X) that satisfy that D(P||Q)<ϵ while H(Q)−H(P)>δ.

The proof of Lemma A1 derives from the same construction presented in the proof of [26] (Theorem 1), i.e., $P=(p_{1},\ldots ,p_{L})$ and a modification of it $Q_{M}=\left(p_{1}\cdot \left(1-1/\sqrt{M}\right),p_{2}+p_{1}/M\sqrt{M},\ldots ,p_{L}+p_{1}/M\sqrt{M},p_{1}/M\sqrt{M},\ldots ,p_{1}/M\sqrt{M}\right)$ both distribution of finite support and consequently in H(X). It is simple to verify that as *M* goes to infinity $D(P||Q_{M})⟶0$ while $H\left(Q_{M}\right)-H\left(P\right)⟶\infty$.

**Proof.** For any pair of distribution *P*, *Q* in H(X), Le Cam’s two point method [53] shows that:

(A1) $$R_{n}^{*}\geq \frac{1}{4}{\left(H(Q)-H(P)\right)}^{2}\exp^{-nD(P||Q)}.$$

Adopting Lemma A1 and Equation (A1), for any *n* and any arbitrary ϵ>0 and δ>0, we have that $R_{n}^{*}>\delta^{2}\exp^{-n\epsilon }/4$. Then exploiting the discontinuity of the entropy in infinite alphabets, we can fix ϵ and make δ arbitrar large. ☐

## Appendix B. Proposition A2

**Proposition** **A2.** *Under the assumptions of Theorem 3:*

(A2) $$\begin{array}{c} \lim_{n\to \infty }\underset{x\in A_{{\tilde{\mu }}_{n}}}{\sup}\left|\frac{d{\tilde{\mu }}_{n}}{d\mu_{n}^{*}}\left(x\right)-1\right|=0,\;P_{\mu }\text{-}a.s. \end{array}$$

**Proof.** First note that $A_{{\tilde{\mu }}_{n}}=A_{\mu_{n}^{*}}$, then $\frac{d{\tilde{\mu }}_{n}}{d\mu_{n}^{*}}\left(x\right)$ is finite and $\forall x\in A_{{\tilde{\mu }}_{n}}$

(A3) $$\frac{d{\tilde{\mu }}_{n}}{d\mu_{n}^{*}}\left(x\right)=\frac{\left(1-a_{n}\right)\cdot \mu \left(A_{n}\left(x\right)\right)+a_{n}v\left(A_{n}\left(x\right)\right)}{\left(1-a_{n}\right)\cdot {\hat{\mu }}_{n}\left(A_{n}\left(x\right)\right)+a_{n}v\left(A_{n}\left(x\right)\right)}.$$

Then by construction,

(A4) $$\begin{array}{c} \underset{x\in A_{{\tilde{\mu }}_{n}}}{\sup}\left|\frac{d{\tilde{\mu }}_{n}}{d\mu_{n}^{*}}\left(x\right)-1\right|\leq \underset{A\in \pi_{n}}{\sup}\frac{\left|{\hat{\mu }}_{n}\left(A\right)-\mu \left(A\right)\right|}{a_{n}\cdot h_{n}}. \end{array}$$

From Hoeffding’s inequality, we have that ∀ϵ>0

(A5) $$\begin{array}{c} P_{\mu }^{n}\left(\underset{A\in \pi_{n}}{\sup}\left|{\hat{\mu }}_{n}\left(A\right)-\mu \left(A\right)\right|>\epsilon \right)\leq 2\cdot \left|\pi_{n}\right|\cdot \exp^{-2n\epsilon^{2}}. \end{array}$$

By condition ii), given that $\left(1/a_{n}h_{n}\right)$ is $o(n^{\tau })$ for some τ∈(0,1/2), then there exists $\tau_{o}\in \left(0,1\right)$ such that

$$\begin{array}{c} \lim_{n\to \infty }\frac{1}{n^{\tau_{o}}}\ln P_{\mu }^{n}\left(\underset{x\in A_{{\tilde{\mu }}_{n}}}{\sup}\left|\frac{d{\tilde{\mu }}_{n}}{d\mu_{n}^{*}}\left(x\right)-1\right|>\epsilon \right)\leq \lim_{n\to \infty }\frac{1}{n^{\tau_{o}}}\ln\left(2\left|\pi_{n}\right|\right)-2\cdot {\left(n^{\frac{1-\tau_{o}}{2}}a_{n}h_{n}\epsilon \right)}^{2}=-\infty . \end{array}$$

This implies that $P_{\mu }^{n}\left(\sup_{x\in A_{{\tilde{\mu }}_{n}}}\left|\frac{d{\tilde{\mu }}_{n}}{d\mu_{n}^{*}}\left(x\right)-1\right|>\epsilon \right)$ is eventually dominated by a constant time ${\left(e^{-n^{\tau_{o}}}\right)}_{n\geq 1}$, which from the Borel-Cantelli Lemma [43] implies that

(A6) $$\begin{array}{c} \lim_{n\to \infty }\underset{x\in A_{{\tilde{\mu }}_{n}}}{\sup}\left|\frac{d{\tilde{\mu }}_{n}}{d\mu_{n}^{*}}\left(x\right)-1\right|=0,\;P_{\mu }\text{-}a.s.. \end{array}$$

☐

## Appendix C. Proposition A3

**Proposition** **A3.**

$$\begin{array}{c} D(\mu_{\epsilon_{n}}\left|\right|{\hat{\mu }}_{n,\epsilon_{n}})\leq \frac{2\log\frac{e}{\epsilon_{n}}}{\mu (\Gamma_{\epsilon_{n}})}\cdot V\left(\mu /\sigma_{\epsilon_{n}},{\hat{\mu }}_{n}/\sigma_{\epsilon_{n}}\right) \end{array}$$

**Proof.** From definition,

(A7) $$\begin{array}{c} D(\mu_{\epsilon_{n}}\left|\right|{\hat{\mu }}_{n,\epsilon_{n}})=\frac{1}{\mu (\Gamma_{\epsilon_{n}})}\sum_{x\in \Gamma_{\epsilon_{n}}}f_{\mu }\left(x\right)\log\frac{f_{\mu }\left(x\right)}{f_{{\hat{\mu }}_{n}}\left(x\right)}+\log\frac{{\hat{\mu }}_{n}\left(\Gamma_{\epsilon_{n}}\right)}{\mu (\Gamma_{\epsilon_{n}})}. \end{array}$$

For the right term in the RHS of (A7):

(A8) $$\begin{array}{c} \log\frac{{\hat{\mu }}_{n}\left(\Gamma_{\epsilon_{n}}\right)}{\mu (\Gamma_{\epsilon_{n}})}\leq \frac{\log(e)}{\mu (\Gamma_{\epsilon_{n}})}\left|{\hat{\mu }}_{n}\left(\Gamma_{\epsilon_{n}}\right)-\mu \left(\Gamma_{\epsilon_{n}}\right)\right|. \end{array}$$

For the left term in the RHS of (A7):

(A9) $$\begin{array}{cc} \left|\sum_{x\in \Gamma_{\epsilon_{n}}}f_{\mu }\left(x\right)\log\frac{f_{\mu }\left(x\right)}{f_{{\hat{\mu }}_{n}}\left(x\right)}\right| & =\left|\sum_{\begin{array}{c} x\in \Gamma_{\epsilon_{n}}\\ f_{\mu }\left(x\right)\leq f_{{\hat{\mu }}_{n}}\left(x\right) \end{array}}f_{\mu }\left(x\right)\log\frac{f_{\mu }\left(x\right)}{f_{{\hat{\mu }}_{n}}\left(x\right)}+\sum_{\begin{array}{c} x\in \Gamma_{\epsilon_{n}}\\ f_{\mu }\left(x\right)>f_{{\hat{\mu }}_{n}}\left(x\right)\geq \epsilon_{n} \end{array}}f_{\mu }\left(x\right)\log\frac{f_{\mu }\left(x\right)}{f_{{\hat{\mu }}_{n}}\left(x\right)}\right|\\ & \leq \sum_{\begin{array}{c} x\in \Gamma_{\epsilon_{n}}\\ f_{\mu }\left(x\right)\leq f_{{\hat{\mu }}_{n}}\left(x\right) \end{array}}f_{\mu }\left(x\right)\log\frac{f_{{\hat{\mu }}_{n}}\left(x\right)}{f_{\mu }\left(x\right)}+\sum_{\begin{array}{c} x\in \Gamma_{\epsilon_{n}}\\ f_{\mu }\left(x\right)>f_{{\hat{\mu }}_{n}}\left(x\right)\geq \epsilon_{n} \end{array}}f_{{\hat{\mu }}_{n}}\left(x\right)\log\frac{f_{\mu }\left(x\right)}{f_{{\hat{\mu }}_{n}}\left(x\right)}\\ & +\sum_{\begin{array}{c} x\in \Gamma_{\epsilon_{n}}\\ f_{\mu }\left(x\right)>f_{{\hat{\mu }}_{n}}\left(x\right)\geq \epsilon_{n} \end{array}}\left(f_{\mu }\left(x\right)-f_{{\hat{\mu }}_{n}}\left(x\right)\right)\cdot \log\frac{f_{\mu }\left(x\right)}{f_{{\hat{\mu }}_{n}}\left(x\right)}\\ & \leq \log e\left[\sum_{\begin{array}{c} x\in \Gamma_{\epsilon_{n}}\\ f_{\mu }\left(x\right)\leq f_{{\hat{\mu }}_{n}}\left(x\right) \end{array}}\left(f_{{\hat{\mu }}_{n}}\left(x\right)-f_{\mu }\left(x\right)\right)+\sum_{\begin{array}{c} x\in \Gamma_{\epsilon_{n}}\\ f_{\mu }\left(x\right)>f_{{\hat{\mu }}_{n}}\left(x\right) \end{array}}\left(f_{\mu }\left(x\right)-f_{{\hat{\mu }}_{n}}\left(x\right)\right)\right] \end{array}$$

(A10) $$\begin{array}{c} +\log\frac{1}{\epsilon_{n}}\cdot \sum_{\begin{array}{c} x\in \Gamma_{\epsilon_{n}}\\ f_{\mu }\left(x\right)>f_{{\hat{\mu }}_{n}}\left(x\right) \end{array}}\left(f_{\mu }\left(x\right)-f_{{\hat{\mu }}_{n}}\left(x\right)\right) \end{array}$$

(A11) $$\begin{array}{c} \leq \left(\log e+\log\frac{1}{\epsilon_{n}}\right)\cdot \sum_{x\in \Gamma_{\epsilon_{n}}}\left|f_{\mu }\left(x\right)-f_{{\hat{\mu }}_{n}}\left(x\right)\right|. \end{array}$$

The first inequality in (A9) is by triangular inequality, the second in (A10) is from the fact that lnx≤x−1 for x>0. Finally, from definition of the total variational distance over $\sigma_{\epsilon_{n}}$ in (59) we have that

(A12) $$\begin{array}{c} 2\cdot V\left(\mu /\sigma_{\epsilon_{n}},{\hat{\mu }}_{n}/\sigma_{\epsilon_{n}}\right)=\sum_{x\in \Gamma_{\epsilon_{n}}}\left|f_{\mu }\left(x\right)-f_{{\hat{\mu }}_{n}}\left(x\right)\right|+\left|{\hat{\mu }}_{n}\left(\Gamma_{\epsilon_{n}}\right)-\mu \left(\Gamma_{\epsilon_{n}}\right)\right|, \end{array}$$

which concludes the argument from (A7)–(A9). ☐

## Appendix D. Proposition A4

**Proposition** **A4.** *Considering that $(k_{n})\to \infty$, there exists K>0 and N>0 such that ∀n≥N,*

(A13) $$V\left({\tilde{\mu }}_{k_{n}},{\hat{\mu }}_{k_{n},n}^{*}\right)\leq K\cdot V\left(\mu /\sigma_{k_{n}},{\hat{\mu }}_{n}/\sigma_{k_{n}}\right).$$

**Proof.** 

(A14) $$\begin{array}{cc} V({\tilde{\mu }}_{k_{n}},{\hat{\mu }}_{k_{n},n}^{*}) & =\frac{1}{2}\sum_{x\in A_{\mu }\cap \Gamma_{k_{n}}}\left|\frac{\mu \left\{x\right\}}{\mu (\Gamma_{k_{n}})}-\frac{{\hat{\mu }}_{n}\left\{x\right\}}{{\hat{\mu }}_{n}\left(\Gamma_{k_{n}}\right)}\right|\\ & \leq \frac{1}{2\mu (\Gamma_{k_{n}})}\left[\sum_{x\in A_{\mu }\cap \Gamma_{k_{n}}}\left|{\hat{\mu }}_{n}\left\{x\right\}-\mu \left\{x\right\}\right|+\sum_{x\in A_{\mu }\cap \Gamma_{k_{n}}}{\hat{\mu }}_{n}\left\{x\right\}\left|\frac{\mu (\Gamma_{k_{n}})}{{\hat{\mu }}_{n}\left(\Gamma_{k_{n}}\right)}-1\right|\right]\\ & =\frac{1}{2\mu (\Gamma_{k_{n}})}\left[2\cdot V\left(\mu /\sigma_{k_{n}},{\hat{\mu }}_{n}/\sigma_{k_{n}}\right)+\left|\mu \left(\Gamma_{k_{n}}\right)-{\hat{\mu }}_{n}\left(\Gamma_{k_{n}}\right)\right|\right]\\ & \leq \frac{3\cdot V(\mu /\sigma_{k_{n}},{\hat{\mu }}_{n}/\sigma_{k_{n}})}{2\mu (\Gamma_{k_{n}})}. \end{array}$$

By the hypothesis $\mu (\Gamma_{k_{n}})\to 1$, which concludes the proof. ☐

## Appendix E. Proposition A5

**Proposition** **A5.** *If $\epsilon_{n}$ is $O(n^{-\tau })$ with τ∈(0,1/2), then*

$$\lim\underset{n\to \infty }{\sup}b_{\epsilon_{n}}\left(X_{1},\ldots ,X_{n}\right)-a_{2\epsilon_{n}}\leq 0,\;P_{\mu }-a.s..$$

**Proof.** Let us define the set

$$B_{n}=\left\{\left(x_{1},\ldots ,x_{n}\right):{\tilde{\Gamma }}_{2\epsilon_{n}}\subset \Gamma_{\epsilon_{n}}\right\}\subset X^{n}.$$

From definition every sequence $\left(x_{1},\ldots ,x_{n}\right)\in B_{n}$ is such that $b_{\epsilon_{n}}\left(x_{1},\ldots ,x_{n}\right)\leq a_{2\epsilon_{n}}$ and, consequently, we just need to prove that $P_{\mu }\left(\lim\inf_{n\to \infty }B_{n}\right)=P_{\mu }\left(\cup_{n\geq 1}\cap_{k\geq n}B_{k}\right)=1$ [42]. Furthermore, if $\sup_{x\in {\tilde{\Gamma }}_{2\epsilon_{n}}}\left|{\hat{\mu }}_{n}\left(\left\{x\right\}\right)-\mu \left(\left\{x\right\}\right)\right|\leq \epsilon_{n}$, then by definition of ${\tilde{\Gamma }}_{2\epsilon_{n}}$ in (65), we have that ${\hat{\mu }}_{n}\left(\left\{x\right\}\right)\geq \epsilon_{n}$ for all $x\in \Gamma_{2\epsilon_{n}}$ (i.e., ${\tilde{\Gamma }}_{2\epsilon_{n}}\subset \Gamma_{\epsilon_{n}}$). From this

(A15) $$\begin{array}{cc} P_{\mu }^{n}\left(B_{n}^{c}\right) & \leq P_{\mu }^{n}\left(\underset{x\in {\tilde{\Gamma }}_{2\epsilon_{n}}}{\sup}\left|{\hat{\mu }}_{n}\left(\left\{x\right\}\right)-\mu \left(\left\{x\right\}\right)\right|>\epsilon_{n}\right)\leq \left|{\tilde{\Gamma }}_{2\epsilon_{n}}\right|\cdot e^{-2n\epsilon_{n}^{2}}\leq \frac{1}{2\epsilon_{n}}\cdot e^{-2n\epsilon_{n}^{2}}, \end{array}$$

from the Hoeffding’s inequality [28,52], the union bound and the fact that by construction $\left|{\tilde{\Gamma }}_{2\epsilon_{n}}\right|\leq \frac{1}{2\epsilon_{n}}$. If we consider $\epsilon_{n}=O\left(n^{-\tau }\right)$ and l>0, we have that:

(A16) $$\begin{array}{c} \frac{1}{n^{l}}\cdot \ln P_{\mu }^{n}\left(B_{n}^{c}\right)\leq \frac{1}{n^{l}}\ln\left(1/2\cdot n^{\tau }\right)-2n^{1-2\tau -l}. \end{array}$$

From (A16) for any τ∈(0,1/2) there is l∈(0,1−2τ] such that $P_{\mu }^{n}\left(B_{n}^{c}\right)$ is bounded by a term $O(e^{-n^{l}})$. This implies that $\sum_{n\geq 1}P_{\mu }^{n}\left(B_{n}^{c}\right)<\infty$, that suffices to show that $P_{\mu }\left(\cup_{n\geq 1}\cap_{k\geq n}B_{k}\right)=1$. ☐

## Appendix F. Auxiliary Results for Theorem 5

Let us first consider the series

(A17) $$\begin{array}{cc} S_{x_{o}}=\sum_{x\geq x_{o}}x^{-p} & =x_{o}^{-p}\cdot \left(1+{\left(\frac{x_{o}}{x_{o}+1}\right)}^{p}+{\left(\frac{x_{o}}{x_{o}+2}\right)}^{p}+\ldots \right)\\ & =x_{o}^{-p}\cdot \left({\tilde{S}}_{x_{o},0}+{\tilde{S}}_{x_{o},1}+\ldots +{\tilde{S}}_{x_{o},x_{o}-1}\right), \end{array}$$

where ${\tilde{S}}_{x_{o},j}\equiv \sum_{k=0}^{\infty }{\left(\frac{k\cdot x_{o}+j}{x_{o}}\right)}^{-p}$ for all $j\in \left\{0,\ldots ,x_{o}-1\right\}$. It is simple to verify that for all $j\in \left\{0,\ldots ,x_{o}-1\right\}$, ${\tilde{S}}_{x_{o},j}\leq {\tilde{S}}_{x_{o},0}=\sum_{k\geq 0}k^{-p}<\infty$ given that by hypothesis p>1. Consequently, $S_{x_{o}}\leq x_{o}^{1-p}\cdot \sum_{k\geq 0}k^{-p}$.

Similarly, for the second series we have that:

(A18) $$\begin{array}{cc} R_{x_{o}}=\sum_{x\geq x_{o}}x^{-p}\log x & =x_{o}^{-p}\cdot \left(\log\left(x_{o}\right)+\left(\frac{x_{o}}{x_{o}+1}\right)\log\left(x_{o}+1\right)+\left(\frac{x_{o}}{x_{o}+2}\right)\log\left(x_{o}+2\right)+\ldots \right)\\ & =x_{o}^{-p}\cdot \left({\tilde{R}}_{x_{o},0}+{\tilde{R}}_{x_{o},2}+\ldots +{\tilde{R}}_{x_{o},x_{o}-1}\right), \end{array}$$

where ${\tilde{R}}_{x_{o},j}\equiv \sum_{k=1}^{\infty }{\left(\frac{k\cdot x_{o}+j}{x_{o}}\right)}^{-p}\cdot \log\left(kx_{o}+j\right)$ for all $j\in \left\{0,\ldots ,x_{o}-1\right\}$. Note again that ${\tilde{R}}_{x_{o},j}\leq {\tilde{R}}_{x_{o},0}<\infty$ for all $j\in \left\{0,\ldots ,x_{o}-1\right\}$, and, consequently, $R_{x_{o}}\leq x_{o}^{1-p}\cdot \sum_{k\geq 1}k^{-p}\log k$ from (A18).

## Appendix G. Proposition A6

**Proposition** **A6.** *If $\epsilon_{n}$ is $O(n^{-\tau })$ with τ∈(0,1/2), then*

$$\lim\underset{n\to \infty }{\inf}\xi_{n}\left(X_{1},\ldots ,X_{n}\right)-\rho_{n}\left(X_{1},\ldots ,X_{n}\right)\geq 0,\;\;P_{\mu }-a.s..$$

**Proof.** By definition if $\sigma \left(\Gamma_{\epsilon_{n}}\right)\subset \sigma \left({\tilde{\Gamma }}_{\epsilon_{n}/2}\right)$ then $\xi_{n}\left(X_{1},\ldots ,X_{n}\right)\geq \rho_{n}\left(X_{1},\ldots ,X_{n}\right)$. Consequently, if we define the set:

(A19) $$B_{n}=\left\{\left(x_{1},\ldots ,x_{n}\right):\sigma \left(\Gamma_{\epsilon_{n}}\right)\subset \sigma \left({\tilde{\Gamma }}_{\epsilon_{n}/2}\right)\right\},$$

then the proof reduced to verify that $P_{\mu }\left(\lim\inf_{n\to \infty }B_{n}\right)$ $=P_{\mu }\left(\cup_{n\geq 1}\cap_{k\geq n}B_{k}\right)=1$. On the other hand, if $\sup_{x\in \Gamma_{\epsilon_{n}}}\left|{\hat{\mu }}_{n}\left(\left\{x\right\}\right)-\mu \left(\left\{x\right\}\right)\right|\leq \epsilon_{n}/2$ then by definition of $\Gamma_{\epsilon }$, for all $x\in \Gamma_{\epsilon_{n}}$ $\mu \left(\left\{x\right\}\right)\geq \epsilon_{n}/2$, i.e., $\Gamma_{\epsilon_{n}}\subset {\tilde{\Gamma }}_{\epsilon_{n}/2}$. In other words,

(A20) $$C_{n}=\left\{\left(x_{1},\ldots ,x_{n}\right):\underset{x\in \Gamma_{\epsilon_{n}}}{\sup}\left|{\hat{\mu }}_{n}\left(\left\{x\right\}\right)-\mu \left(\left\{x\right\}\right)\right|\leq \epsilon_{n}/2\right\}\subset B_{n}.$$

Finally,

(A21) $$\begin{array}{cc} P_{\mu }^{n}\left(C_{n}^{c}\right) & =P_{\mu }^{n}\left(\underset{x\in \Gamma_{\epsilon_{n}}}{\sup}\left|{\hat{\mu }}_{n}\left(\left\{x\right\}\right)-\mu \left(\left\{x\right\}\right)\right|>\epsilon_{n}/2\right)\leq \left|\Gamma_{\epsilon_{n}}\right|\cdot e^{-n\epsilon^{2}/2}\leq \frac{1}{\epsilon_{n}}\cdot e^{-n\epsilon^{2}/2}. \end{array}$$

In this context, if we consider $\epsilon_{n}=O\left(n^{-\tau }\right)$ and l>0, then we have that:

(A22) $$\begin{array}{c} \frac{1}{n^{l}}\cdot \ln P_{\mu }^{n}\left(C_{n}^{c}\right)\leq \tau \cdot \frac{\ln n}{n^{l}}-\frac{n^{1-2\tau -l}}{2}. \end{array}$$

Therefore, we have that for any τ∈(0,1/2) we can take l∈(0,1−2τ] such that $P_{\mu }^{n}\left(C_{n}^{c}\right)$ is bounded by a term $O(e^{-n^{l}})$. Then, the Borel Cantelli Lemma tells us that $P_{\mu }\left(\cup_{n\geq 1}\cap_{k\geq n}C_{k}\right)=1$, which concludes the proof from (A20). ☐

## Appendix H. Proposition A7

**Proposition** **A7.** *For the p-power tail dominating distribution stated in Theorem 5, if* $\left(\epsilon_{n}\right)$ *is* $O(n^{-\tau })$ *with* τ∈(0,p) *then* $\xi_{n}\left(X_{1},\ldots ,X_{n}\right)\;\mathrm{is}\;o\left(n^{-q}\right)\;\mathrm{for}\;\mathrm{all}\;q\in \left(0,\left(1-\tau /p\right)/2\right)$*,* $P_{\mu }$*-a.s.*

**Proof.** From the Hoeffding’s inequality we have that

(A23) $$\begin{array}{cc} P_{\mu }^{n}\left(\left\{x_{1},\ldots ,x_{n}:\xi_{n}\left(x_{1},\ldots ,x_{n}\right)>\delta \right\}\right) & \leq \left|\sigma ({\tilde{\Gamma }}_{\epsilon_{n}/2})\right|\cdot e^{-2n\frac{\delta^{2}}{\log{\left(1/\epsilon_{n}\right)}^{2}}}\\ & \leq 2^{{\left(\frac{2k_{o}}{\epsilon_{n}}\right)}^{1/p}+1}\cdot e^{-2n\frac{\delta^{2}}{\log{\left(1/\epsilon_{n}\right)}^{2}}}, \end{array}$$

the second inequality using that ${\tilde{\Gamma }}_{\epsilon }\leq {\left(\frac{k_{0}}{\epsilon }\right)}^{1/p}+1$ from the definition of ${\tilde{\Gamma }}_{\epsilon }$ in (65) and the tail bounded assumption on μ. If we consider $\epsilon_{n}=O\left(n^{-\tau }\right)$ and l>0, then we have that:

(A24) $$\begin{array}{cc} \frac{1}{n^{l}}\cdot \ln P_{\mu }^{n}\left(\left\{x_{1},\ldots ,x_{n}:\xi_{n}\left(x_{1},\ldots ,x_{n}\right)>\delta \right\}\right) & \leq \ln2\cdot \left(Cn^{\tau /p-l}+n^{-l}\right)-\frac{2\delta^{2}}{\tau^{2}}\cdot \frac{n^{1-l}}{\log n^{2}} \end{array}$$

for some constant C>0. Then in order to obtain that $\xi_{n}\left(X_{1},\ldots ,X_{n}\right)$ converges almost surely to zero from (A24), it is sufficient that l>0, l<1, and l>τ/p. This implies that if τ<p, there is l∈(τ/p,1) such that such that $P_{\mu }^{n}\left(\xi_{n}\left(x_{1},\ldots ,x_{n}\right)>\delta \right)$ is bounded by a term $O(e^{-n^{l}})$ and, consequently, $\lim_{n\to \infty }\xi_{n}\left(X_{1},\ldots ,X_{n}\right)=0$, $P_{\mu }$-a.s. (this by using the same steps used in Appendix G). Moving to the rate of convergence of $\xi_{n}\left(X_{1},\ldots ,X_{n}\right)$ (assuming that τ<p), let us consider $\delta_{n}=n^{-q}$ for some q≥0. From (A24):

(A25) $$\begin{array}{cc} \frac{1}{n^{l}}\cdot \ln P_{\mu }^{n}\left(\left\{x_{1},\ldots ,x_{n}:\xi_{n}\left(x_{1},\ldots ,x_{n}\right)>\delta_{n}\right\}\right) & \leq \ln2\cdot \left(Cn^{\tau /p-l}+n^{-l}\right)-\frac{2\delta^{2}}{\tau^{2}}\cdot \frac{n^{1-2q-l}}{\log n^{2}}. \end{array}$$

To make $\xi_{n}\left(X_{1},\ldots ,X_{n}\right)$ being $o(n^{-q})$ P-a.s., a sufficient condition is that l>0, l>τ/p, and l<1−2q. Therefore (considering that τ<p), the admissibility condition on the existence of a exponential rate of convergence $O(e^{-n^{l}})$ for l>0 for the deviation event $\left\{x_{1},\ldots ,x_{n}:\xi_{n}\left(x_{1},\ldots ,x_{n}\right)>\delta_{n}\right\}$ is that τ/p<1−2q, which is equivalent to $0<q<\frac{1-\tau /p}{2}$. ☐
